# Inflammation, Oxidative Stress, Senescence in Atherosclerosis: Thioredoxine-1 as an Emerging Therapeutic Target

**DOI:** 10.3390/ijms23010077

**Published:** 2021-12-22

**Authors:** Khadija El Hadri, Rémy Smith, Eric Duplus, Chahrazade El Amri

**Affiliations:** Biological Adaptation and Ageing (B2A), CNRS UMR 8256, INSERM U1164, F-75252, Institut de Biologie Paris Seine (IBPS), Sorbonne Université, 75005 Paris, France; remy.smith@sorbonne-universite.fr (R.S.); eric.duplus@sorbonne-universite.fr (E.D.)

**Keywords:** atherosclerosis, inflammation, senescence, oxidative stress, thioredoxin, antioxidants, senomodulators, anti-inflammatory agents

## Abstract

Atherosclerosis is a leading cause of cardiovascular diseases (CVD) worldwide and intimately linked to aging. This pathology is characterized by chronic inflammation, oxidative stress, gradual accumulation of low-density lipoproteins (LDL) particles and fibrous elements in focal areas of large and medium arteries. These fibrofatty lesions in the artery wall become progressively unstable and thrombogenic leading to heart attack, stroke or other severe heart ischemic syndromes. Elevated blood levels of LDL are major triggering events for atherosclerosis. A cascade of molecular and cellular events results in the atherosclerotic plaque formation, evolution, and rupture. Moreover, the senescence of multiple cell types present in the vasculature were reported to contribute to atherosclerotic plaque progression and destabilization. Classical therapeutic interventions consist of lipid-lowering drugs, anti-inflammatory and life style dispositions. Moreover, targeting oxidative stress by developing innovative antioxidant agents or boosting antioxidant systems is also a well-established strategy. Accumulation of senescent cells (SC) is also another important feature of atherosclerosis and was detected in various models. Hence, targeting SCs appears as an emerging therapeutic option, since senolytic agents favorably disturb atherosclerotic plaques. In this review, we propose a survey of the impact of inflammation, oxidative stress, and senescence in atherosclerosis; and the emerging therapeutic options, including thioredoxin-based approaches such as anti-oxidant, anti-inflammatory, and anti-atherogenic strategy with promising potential of senomodulation.

## 1. Introduction

Cardiovascular diseases (CVDs), principally ischemic heart disease and stroke, are the leading cause of global mortality and a major contributor to disability. Prevalent cases of total CVD nearly doubled from 271 million in 1990 to 523 million in 2019, and the number of CVD deaths continuously increased from 12.1 million in 1990, reaching 18.6 million in 2019. Globally in 2019, CVD was the underlying cause of >6 million deaths occurring between the ages of 30 and 70 years, approximately one third of all deaths globally [1].

The CVDs are often complications of atherosclerosis, characterized by the formation of fibrofatty lesions in the artery wall that begins early in life and progresses gradually, remaining usually asymptomatic for a long period of time [2]. Atherosclerosis is a disease characterized by low-grade, chronic inflammation of the arterial wall. The formation of atherosclerotic plaque is due to the accumulation of apolipoprotein B-containing low-density lipoprotein (LDL) particles and fibrous elements in focal areas of large and medium arteries [3]. The risk factors for CVD can be either non-modifiable; such as age, gender, ethnicity, and genetics; or modifiable, such as elevated serum lipids, high blood pressure, high fasting plasma glucose, high LDL-cholesterol, low physical activity, obesity, ambient and household air pollution, and tobacco [1,4,5].

This review aims to summarize our current understanding of inflammatory and oxidative processes, and cell senescence in atherosclerosis. We then particularly discuss the mechanisms of inflammation/resolution that become impaired in atherosclerosis and that are potentially amenable to therapeutic intervention by resolution-mediator therapy. In particular, we highlight the roles of thioredoxine-1 system as an endogenous resolution mediator in suppressing atherogenic processes and how this system might be used as a therapy to prevent atherosclerotic cardiovascular diseases.

## 2. Atherosclerosis, Pathophysiological Process

### 2.1. General Features

The healthy artery wall consists of three morphologically distinct layers. The tunica intima, the innermost layer, is bounded by a monolayer of endothelial cells (EC) on the luminal side and a sheet of elastic fibers, the internal elastic lamina, on the peripheral side. The normal intima is a very thin region and consists of extracellular connective tissue matrix, primarily proteoglycans and collagen. The tunica media, the middle layer, consists of quiescent smooth muscle cells (SMCs) and a well-organized extracellular matrix comprising elastin, collagen and other macromolecules. The outermost layer, tunica adventitia, consists of nerve endings, mast cells, and micro-vessels that nourish the outer layer of the media. Adventitial resident macrophages contribute to the physiology and diameter of the vessel wall via cross-talk with SMCs. These vascular macrophages are largely derived from embryonic precursor cells and self-renewal in situ [6,7].

The endothelium, with its intercellular tight junctional complexes, functions as a selectively permeable barrier between blood and the underlying tissues. It has sensory, autocrine, paracrine, and endocrine functions that are highly relevant to vascular homeostasis [8]. ECs mediate several functions, including modulation of vascular tone, regulation of neovascularization, inflammation and immune response. ECs metabolize L-arginine via the endothelial isoform of nitric oxide synthase (eNOS), in NADPH and cofactor tetrahydrobioprotein (BH4) dependent manner, to form nitric oxide (NO) that can rapidly diffuse across cell membranes to act as a potent paracrine mediator, but it can also react with superoxide (O_2_^•^−) to form peroxynitrite anion (ONOO−), which leads to its inactivation. The actions of NO on adjacent SMCs and circulating blood platelets and leukocytes are particularly relevant for vascular homeostasis [9].

One of the earliest events in atherosclerosis is the impairment of endothelium, a complex pathophysiological process, including both the activation of the ECs and the onset of endothelial dysfunction [10]. In cardiovascular medicine, the term “endothelial dysfunction” is typically used to refer to abnormalities in the production or bioavailability of endothelial-derived NO and resultant deleterious changes in vascular reactivity. Activation of ECs occurs under irritating stimuli (such as dyslipidemia, hypertension, disturbed shear stress (low, turbulent, or oscillatory shear stress) or pro-inflammatory mediators) and enhances secretion of chemokines and expression of cell surface adhesion molecules that attract and bind monocytes [10,11]. Parallel changes in endothelial permeability and the composition of the extracellular matrix beneath the endothelium promote the entry and retention of LDL particles in the artery wall [12]. The interaction of LDL with intimal extracellular proteoglycans inhibits the movement of the lipoproteins, thereby resulting in much higher subendothelial concentrations of these particles in the intima than in any other tissue [13]. LDL undergo numerous modifications that give the atherogenic properties to the particles. Among such modifications, there are desialylation, oxidation, and changes in size and density of LDL [14]. These processes are followed by entry of the monocytes into the subendothelial space where they differentiate into macrophages. Macrophages express on their surface scavenger receptors (SR) such as CD36, SR-A1, and lectin-like oxidized LDL (oxLDL) receptor-1 (LOX-1) that bind to oxLDL, allowing the uptake of these proteins into the cell [15]. OxLDL particles bound to scavenger receptors, are phagocytosed by macrophages, and metabolized into cholesterol esters and then into free fatty acids and cholesterol [15]. Free cholesterol is stored and/or carried outside the cell by the ATP-binding cassette transporters ABCA1 and ABCG1 and the scavenger receptor SR-BI. However, this regulation of cholesterol metabolism is altered in atherosclerosis; in this sense, foam cells are the result of an unregulated accumulation of oxLDL and cholesterol esters within the macrophages located in the intima in response to activated ECs by inflammation. To a lesser degree, foam cells are derived from transformed SMCs [3,15,16,17]. 

Macrophages in atherosclerotic lesions mainly originate from circulating monocytes, which migrate into atherosclerosis-susceptible sites of the arterial wall and into the growing atherosclerotic lesions [18,19]. Local proliferation of macrophages and trans-differentiation of intimal SMCs into macrophage-like cells are two additional sources of macrophages in the lesions [20]. Intimal SMCs not only accumulate cholesterol and turn into foam cells but the cholesterol accumulation in the cell can also drive their switch into pro-inflammatory, macrophage-like cells [9,21].

The foam cells secrete inflammatory cytokines, reactive oxygen species (ROS) and other mediators contributing to SMC migration from the media. In the intima, the resident and recruited SMCs produce extracellular matrix molecules, including interstitial collagen and elastin, and form a fibrous cap that covers the plaque [3,22]. This cap typically overlies a collection of macrophage-derived foam cells, some of which can die, especially by apoptosis, and release lipids that accumulate extracellularly. The inefficient clearance of dead cells, a process known as efferocytosis, can promote the accumulation of cellular debris and extracellular lipids, forming a lipid-rich pool called the necrotic core of the plaque [12].

In this inflammatory microenvironment, activated macrophages show increased production of the matrix metalloproteinases (MMPs) family that degrade the extracellular matrix constituents contributing to thinning and structural weakening of the fibrous cap and increasing the susceptibility of the plaque to rupture. In the worst-case scenario, an occlusive thrombus forms, leading to acute oxygen and nutrient deprivation of distal tissues fed by the artery. When these events occur in the coronary arteries, the region of heart muscle tissue fed by the involved artery becomes injured, and the result is unstable angina, myocardial infarction, or sudden cardiac death [3,12,23].

### 2.2. Inflammation in Atherosclerosis

For a long time, atherosclerosis has been merely considered as a result of lipid accumulation that obstructs arterial vessel wall [24]. Two centuries ago, Virchow highlighted inflammation as a central cause of atherosclerosis [25]. After that and through the discovery of various inflammatory markers, the inflammation has been proposed as a response to vascular injury [26,27]. Hence, atherosclerosis is now defined as a chronic inflammatory disorder, in which inflammation characterizes all phases of the pathogenic process including formation, progression, and rupture of atherosclerotic plaques [28,29,30,31]. Biomarkers of inflammation, notably C-reactive protein, rise in tandem with many established cardiovascular risk factors hence prospectively predicting cardiovascular risk [32].

Atherosclerotic plaque tends to form at sites of flow disturbance. The local hemodynamic environment disturbs homeostatic atheroprotective functions of the endothelium, notably reversing its anti-inflammatory properties [33]. The finding that pro-inflammatory cytokines such as interleukin (IL)-1β and tumor necrosis factor-α (TNF-*α*) could give rise to the expression of adhesion molecules and chemokines by ECs providing a support of the inflammatory basis of atherosclerosis. Under pro-inflammatory stimuli, production of these cytokines in vascular walls may induce the recruitment and accumulation of leukocytes within nascent and growing lesions [18,33,34,35]. The abundance of the monocytes in the circulation, particularly CD14^++^ subpopulation in humans and Ly6C^hi^ (highly expressing the cell surface marker Ly6c) subpopulation in mice, is strongly correlated with atherosclerosis development [36,37]. Ly6C^hi^ monocytes appear to accumulate early in atherosclerotic plaque. They can differentiate into macrophage expressing scavenger receptors, such as SR-A/B and CD36, with high capacity to internalize lipids and become foam cells [38]. Inflammation and lipid accumulation are considered to be key processes of atherosclerosis development [39].

#### 2.2.1. NLRP3 Inflammasome Implication

Accumulation of lipids in macrophages readily induces activation of a multiprotein complex termed the inflammasome [40,41,42]. Of the different inflammasomes, NLRP3 is the most widely studied and a critical regulator notably involved in the pathogenesis of CVDs. Human atherosclerotic lesions show increased expression of the major components of NLRP3 [43], and inhibition of the NLRP3 inflammasome reduces atherosclerosis in Apoe^-^−/− and ApoE2-Ki mice [44,45]. The inflammasome generally requires two signals to assemble and act [46]. Signal 1 primes, via NF-*κ*B pathway, the transcription of the pro-IL-1β and NLRP3 inflammasome constituents, and signal 2, either by the same and/or additional stimuli, activates the inflammasome that recruits the adapter molecule ASC (apoptosis-associated speck-like protein containing a caspase recruitment domain), which links NLRP3 to pro-caspase-1. The association of multiprotein complex activates caspase-1, which catalyzes the cleavage of inactive pro-IL-1β to produce the major pro-inflammatory cytokine IL-1β [47,48,49]. Human atherosclerotic plaques harbor activated inflammasomes [50].

OxLDL has been shown both to prime and to activate the NLRP3 inflammasome in macrophages in mouse models of atherosclerosis. First, oxLDL primes the NLRP3 inflammasome and, then, after having been transported to lysosomes, the oxLDL activates the NLRP3 inflammasome via induction of cholesterol crystallization and ensuing lysosomal damage in the macrophage [51,52,53,54,55]. In addition, other atherosclerosis-relevant stimuli, such as disturbed blood flow, can augment NLRP3 inflammasome activation [56,57]. The local production of IL-1β is central in mediating the proinflammatory response resulting in activation of secondary inflammatory mediators, including IL-6. IL-1β can be consequently considered as both a local vascular and systemic major contributor to atherosclerosis and its complications. IL-1β has multiple inflammatory effects on vascular ECs, SMCs, and macrophages [49]. For example, IL-1β induces adhesion molecules in human ECs [58]; induces autocrine production of platelet-derived growth factor that can stimulate SMC proliferation [59] and the cytokine activates cells involved in innate immunity, particularly macrophages [60].

#### 2.2.2. Implications of Immune Cells

Technical advances (advent of monoclonal antibody, single cell gene expression, and mass spectrometry-based protein expression) has enabled the precise identification of different cell types involved in atherosclerosis. Among the leukocyte population in atherosclerotic plaques, the macrophage, the major cell of innate immunity, dominates. Macrophages have high phenotypic plasticity and undergo a variety of phenotypes/functions depending on number of factors, including local environment (tissue niche); intracellular energy metabolism; and genetic and epigenetic factors [61,62,63,64]. These factors program macrophages for a palette of functional subtypes, in experimental and human atherosclerosis, from inflammatory host defense (M1) to resolution and repair (M2) [38]. Macrophages at both ends of the inflammation-resolution spectrum accumulate during atherosclerotic lesion development [65]. The relative proportion of macrophages with different subtypes varies depending on plaque region. Inflammatory M1 macrophages are enriched in advanced prone to rupture plaque where they secrete inflammatory cytokines and matrix metalloproteinases (MMP-2 and MMP-9) participating to fibrous cap thinning, plaque necrosis and morphological changes that can trigger plaque rupture and luminal thrombosis [66]. Conversely, resolving M2 macrophages function to clear apoptotic cells (efferocytosis), to phagocytose debris, to secrete collagen that can form a protective scar over the lesion, and to produce inflammation-resolving molecules that quell inflammation and promote tissue repair [67,68]. Macrophages play a key role in atherogenesis through their proinflammatory action, which involves the production of IL-1β and TNF-*α*, and following more specific adaptive responses mediated by T cells [69,70].

The initial innate immune response is followed by an antigen-specific adaptive immune response involving different types of T and B cells [71]. T helper 1 (Th1) is the most frequent T cell involved in the atherosclerotic process. Macrophage-derived IL-12 and IL-18 induce Th1 cell differentiation, responding to oxLDL stimuli by secreting further TNF-*α* and interferon γ (IFN-γ), a powerful inductor of atherosclerosis at the different stages of the process [71,72]. Th2 plays a minor role, but it seems to be protective, secreting interleukins that inhibit Th1 cells and induce M2 anti-inflammatory macrophages. Regulatory T (Treg) cells act as atheroprotective cells by secreting IL-10 and transforming growth factor β (TGF-β), playing an immunomodulatory role [72]. In the atherogenic process, Th1 cells increase whereas Treg progressively decreases [64]. Inflammation and immunity are actively involved in the genesis and complications of atherosclerosis [73,74] and inflammatory biomarkers are independent risk factors for cardiovascular events [74].

Of note, in human atherosclerotic plaques, the localization of pro-inflammatory macrophages and resolving macrophages in distinct areas probably reflects, among other things, differences in the composition of macrophage-polarizing factors and cytokines in the respective microenvironments within the plaque [75]. The phenotypic heterogeneity of macrophage subtypes allows macrophages to have various combinations of both pathogenic and protective functions in humans. Moreover, individual macrophages hold the potential for a dynamic phenotype switch, which affects the functional properties of the macrophage [76,77]. Macrophages are in consequence a meaningful target for therapeutic strategies in CVDs.

### 2.3. Oxidative Stress in Atherosclerosis

Oxidative stress was shown to be associated with various human diseases, including cancers, neurodegenerative diseases, chronic inflammation, and cardiovascular disorders. Moreover, major known risk factors of atherosclerosis, including dyslipidemia, diabetes, and hypertension are all associated with oxidative stress [78,79]. Since the 1950s, oxidative modifications of lipids and proteins have been detected in vascular lesions and the degree of oxidation correlates with the severity of disease [80] indicating a role of oxidative stress in atherogenesis. Oxidative stress is characterized by an imbalance between the oxidant and antioxidant systems, resulting in an increase of ROS. We found an increase in the level of free radical peroxidation products and decrease in the activity of antioxidant enzymes in the tissues of animals with experimental atherosclerosis. Similar changes were found in the blood of patients with atherosclerosis and aortic autopsy material with atherosclerotic lesions [81,82].

The vascular wall has oxidant systems such as xanthine oxidase [83], mitochondrial respiratory chain enzymes [78], lipoxygenases [82], uncoupled eNOS [84], NADPH oxidases (Nox) [85], and antioxidant systems, including superoxide dismutase (SOD), catalase, glutathione peroxidases, thioredoxin system, paraoxonases, and peroxiredoxins [79,86,87]. ROS include both free radical and non-free radical chemically active compounds that contain oxygen, among them hydrogen peroxide (H_2_O_2_), superoxide anion (O_2_^•^−), and the hydroxyl radical (OH^•^). Under physiological conditions, ROS are produced at moderate concentrations and play an important role in cell signaling, regulation of cell cycle, apoptosis and gene expression through interaction with transcription factors. Moreover, ROS are generated by phagocytes that use them to kill pathogens and combat infection [88].

One of the main consequences of oxidative stress at the vascular level is endothelial dysfunction. ROS exert their actions mainly via NF-*κ*B, which induces the synthesis of proinflammatory cytokines, such as TNF-*α*, which in turn activate NF-*κ*B [89]. Hence, due to the synergy between ROS and cytokines, ECs promote the synthesis of inflammatory factors and upregulate the expression of adhesion molecules, thus allowing monocytes to transmigrate into the arterial intima [90]. At this early stage of atherosclerosis, Nox is the main source of ROS at the vascular wall [91]. It reduces O_2_ to superoxide anion O_2_^•^−, which in turn interacts with NO to generate the very potent oxidant peroxynitrite ONOO−, reducing the NO bioavailability and leading to endothelial dysfunction [79,85]. Mitochondrial respiratory chain enzyme dysfunction leads to an increased ROS production. Experiments involving the deletion of antioxidant systems in ApoE−/− mice suggest a role for mitochondrial ROS in atherogenesis [92,93,94].

The increased presence of the immune cells in the arterial wall leads to cytokines release and the induction of the inflammatory processes and ROS production. For instance, TNF-*α* was shown to increase mitochondrial ROS production; IL-1β to induce ROS production by Nox; and IFN-γ to induce ROS through both mitochondrial and Nox pathways [95,96]. Therefore, atherosclerosis plaque development is a result of the production and release of both growth factors and ROS. Moreover, ROS can enhance the expression of scavenger receptors on vascular SMCs therefore inducing their ability to internalize and accumulate lipids and transform into foam cells. The release of matrix MMPs, which are responsible for plaque disruption, is also stimulated by ROS. Cyclic strain-induced MMP-2 expression on vascular SMCs was shown to be dependent on Nox activation [97]. In atherosclerosis, deficiency of antioxidant systems can promote disease progression through oxidative stress. For instance, these processes were observed in atherosclerosis models, such as Apoe−/− atherosclerotic mice deficient in superoxide dismutase 2 (SOD 2) [98].

### 2.4. Cellular Senescence in Atherosclerosis

As an age-related disease, atherosclerosis is associated with a number of biological processes including cellular senescence [99]. Moreover, there is extensive evidence of accelerated biological aging in atherosclerosis, with the large majority of the hallmarks of aging being present in advanced plaques. Advanced human atherosclerotic plaques contain p16*^INK4a^* positive cells associated with both vascular SMC and macrophage markers [100,101,102]. However, cellular aging is not just a marker of disease, but contributes directly to atherogenic process [103,104,105,106].

**Figure 1:**
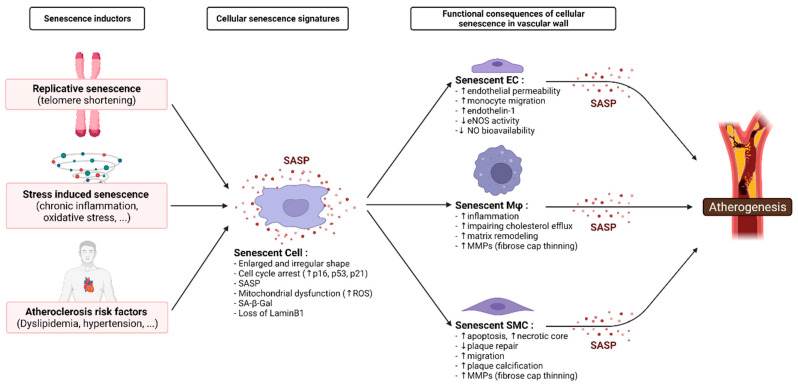
Role of senescence mediated-processes on the physiopathological events of atherosclerosis. Focus on vascular wall senescent cell populations; namely endothelial cells (EC), macrophages (Mφ), and smooth muscle cells (SMC).

Cellular senescence can be a consequence of replicative exhaustion resulting from chronological age or intense proliferation, so called telomere-dependent senescence [107,108,109]. Moreover, exposure to cardiovascular risk factors lead to a stress-induced senescence [110,111,112,113]. Therefore, cellular senescence burden is rather a consequence of a combined effect of chronological aging and risk factor exposure. Being a shared consequence of the effect of all of these various factors, senescence is an important upstream effector that promotes atherogenesis (Figure 1).

The molecular pathways of senescence result in morphological alterations. Senescent cells (SC) are enlarged and have an irregular shape; their nuclear integrity is compromised due to the loss of lamin B1, which also leads to the appearance of cytoplasmic chromatin fragments; they have an increased lysosomal content leading to an increased senescence-associated β-galactosidase activity (SA-β-gal); and they have large but dysfunctional mitochondria that produce high levels of ROS [114]. In addition, SCs are classically characterized by an irreversible cell cycle arrest that is primarily imposed by an upregulation of the cell cycle inhibitors p16*^INK4a^*, p21 and p53 [115,116,117]. SCs exhibit a specific secretory phenotype, namely the senescence-associated secretory phenotype (SASP), that consists of a large wide range of biologically active molecules, inflammatory cytokines, chemokines, growth factors, and proteases [118,119,120]. The SASP is, to a large extent, a transcriptional program mediated by the proinflammatory transcription factor NF-κB [121].

As tissular SCs increase in number, inflammaging, a state of chronic, systemic, low-grade inflammation, is established [122]. The senescence of multiple cell types present in the vasculature were reported to trigger various pathophysiological processes in atherosclerosis, particularly SASP that gradually contributes to atherosclerotic plaque progression and destabilization [105]. Consistent SASP elements in the majority of SCs include IL-1α, IL-1β, IL-6, IL-8, IL-18, and TNF-*α* [112,118,123], all of which are clinically validated as CVD risk factors [124,125]. These inflammatory cytokines promote senescence locally in a paracrine manner [126]. Mounting studies have demonstrated the accumulation of SCs in atherosclerotic lesions from both experimental model and human plaques, providing insights into the association between cellular senescence and plaque progression [127]. This notion is supported by recent results showing that life-long elimination of senescent cells can prevent the development of certain age-related pathologies in a mouse model of segmental accelerated aging, strongly supporting the idea that SCs can be deleterious [128,129].

Clinical evidence of the involvement of cellular senescence in the atherosclerotic vessel wall, in the general population, comes from post-mortem histological analysis that showed that senescent EC and VSMC accumulate substantially more in atherosclerotic than in physiologically aged healthy arteries [100,130,131,132,133]. Expression of senescence marker p16*^INK4a^* in the diseased human coronary arteries positively coincided with unstable plaques and correlated with intra-plaque TNF-*α* levels [102]. Furthermore, coronary vessels from ischemic heart disease patients showed significant endothelial senescent cell burden, while the mostly plaque-free internal mammary arteries from the same donors had no evidence of senescence [130]. In human carotid artery atherosclerosis, senescent SMCs were associated with phenotypical features of plaque instability [100,134,135,136] and accounted for 18% of all plaque cells [100].

The senescence of ECs directly compromised the endothelial barrier through disruption of cellular proliferation, permeability, and motility [137,138,139], possibly contributing to endothelial erosion and intraplaque hemorrhage. Furthermore, senescent ECs show attenuated endothelial NO production [140,141]. NO produced by eNOS normally maintains vascular SMCs in a nondividing, contractile state and suppresses thrombogenic and inflammatory signaling in endothelium. Thus, EC senescence is associated with loss of EC function and a shift towards a pro-inflammatory state, predicted to enhance monocyte migration into the vessel wall [142].

Vascular SMCs in human plaques or derived from plaques show reduced proliferation, early senescence, and increased susceptibility to apoptosis [143]. These properties would reduce the ability to repair plaques that undergo rupture. Aged rodent aortas also show increased levels of IL-6 and aged aortic SMCs have a higher basal secretion of IL-6 than young SMCs as part of the SASP. Moreover, aged SMCs exhibit upregulation of chemokines (CCL2), adhesion molecules (e.g., ICAM-1), and innate immune receptors (e.g., Toll-like receptor 4) [144]. Gardner et al. [145] showed that senescent human vascular SMCs released multiple high-level pro-inflammatory proteins, which might upregulate inflammasome components and increase the metabolic burden of senescent SMCs. These properties generate a proinflammatory environment, further promoting migration of inflammatory cells. Vascular SMC senescence was associated with necrotic core enlargement [134] and plaque calcification [146] in human atherosclerosis. Plaque destabilization through fibrose cap thinning is promoted by various MMPs [147] as MMP-1, -2, -3, -7, -8, -9, -10, -12, -13, and -14 [145,147,148], which are secreted as part of the SASP of senescent SMCs, monocytes, macrophages, and foam cells. Chen Chi et al., recently addressed the relevance of senescent vascular SMCs to atherosclerosis, as well as the potential mechanisms responsible for SMC senescence in these age-related diseases [149].

Both innate and adaptive immune function declines with age [150,151,152], and this contributes to increased susceptibility to sepsis and inflammatory diseases [153]. Franceschi et al. proposed that macrophages play a central role in producing inflammaging, which ultimately impairs the immune response [122]. Senescent macrophages may influence the development of atherosclerosis by impairing cholesterol efflux and promoting inflammatory response. Foamed macrophages with senescence markers coexist with inflammatory cytokines, chemokines, and metalloproteinases during atherosclerosis [104]. In addition, recent studies have reported that a significant proportion of p16*^INK4a^*/SA-β-gal-positive cells accumulating in aging mice are macrophages [154], which have the same right as senescent cells to be considered a possible contributor to aging and his associated pathologies [155].

Additional studies are needed to characterize quantification of SCs, plaque features, and correlation to clinical data to strengthen the causal link between arterial wall cellular senescence and atherosclerosis. In summary, chronic SCs accumulate over time as a result of repeated tissue damage. Through the SASP, cellular senescence exerts many pro-atherogenic effects and it is possibly a key etiologic driver of pathological vascular remodeling, forming a perpetual loop that chronically amplifies the effects of risk factor exposure. Therefore, senescence appears to be a therapeutic target worth exploring for the prevention or treatment of atherosclerosis.

## 3. Thioredoxin as an Emerging Therapeutic Agent

### 3.1. Thioredoxin System

**Figure 2:**
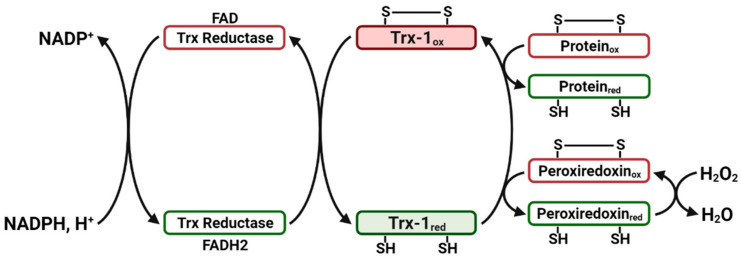
The Redox-Cycling Reactions of the Thioredoxin System. The dithiol moieties of Trx-1 (S-S form) are reduced by receiving electrons from NADPH in the presence of thioredoxin reductase (TrxR). Reduced Trx-1 (SH form) in turn reduces oxidized proteins with disulfide bonds through thiol disulfide exchange reactions. Furthermore, Trx-1 can scavenge free radicals indirectly through peroxiredoxin [157]. Flavin Adenine Dinucleotide (FAD); Nicotinamide Adenine Dinucleotide Phosphate (NADPH_2_).

In 1964, thioredoxin (Trx) was first isolated from *Escherichia coli* by Laurent et al. reporting that *E. coli* also contained an enzyme, thioredoxin-reductase (TrxR), which catalyzes its reduction [156]. There are three distinct forms of human Trx, encoded by separate genes, cytosolic Trx (Trx-1), mitochondrial Trx (Trx-2), and a Trx variant that is highly expressed in spermatozoa (SpTrx/Trx-3) [157,158,159]. Trx-1, the most studied Trx protein, contains a conserved redox catalytic site Cys^32^-Gly-Pro-Cys^35^, which reduces disulfide bonds (-S-S-) of substrate proteins through thiol (SH)/disulfide exchange reaction [160]. The dithiol moieties of Trx-1 are reduced by receiving electrons from Nicotinamide Adenine Dinucleotide Phosphate (NADPH) in the presence of TrxR. Reduced Trx in turn reduces proteins with disulfide bonds by transferring electrons from its reactive cysteines through thiol disulfide exchange reactions (Figure 2). Trx-1 is negatively regulated by thioredoxin-interacting protein (TXNIP), which binds to the reduced form of Trx-1 and blocks its functions [161]. Thus, Trx-1, NADPH, TrxR, and TXNIP are collectively termed the Trx system [162,163].

In addition to the two cysteine residues in the active-site (Cys^32^, Cys^35^), human Trx-1, but not bacterial Trx, contain three other, critical structural cysteines residues; Cys^62^, Cys^69^, and Cys^73^, providing unique biological properties to Trx-1. These cystine residues can undergo post-translational modifications having a significant effect on Trx-1 function [164,165,166]. Both Cys^62^ and Cys^69^ are sites of S-nitrosylation, whereas Cys^73^ is a multimodification site, undergoing S-nitrosylation, glutathionylation, dimerization, or 4- hydroxy-2-nonenal (HNE) modification [157].

Among the main thiol compounds of biological interest, Trx-1 functions as an antioxidant through its facilitation of the reduction of other thiol-containing proteins via the cysteine thiol-disulfid exchange. Thiol is a highly active form of reduced sulphur in amino acids such as cysteine (Cys) in peptides (e.g., glutathione), and proteins (e.g., Trx), and is particularly sensitive to redox reactions, acts as a major redox sensor as well as a switch that modifies function and interactivity of proteins. As such, the dithiol Trx-1 plays a vital role in maintaining the cellular redox homeostasis. Depending on its subcellular localization, Trx-1 exerts different roles. It can be found in the extracellular environment, cytoplasm, and the nucleus [157]. In the extracellular environment, Trx-1 exhibits chemokine-like activity [158], while in the cytoplasm, it regulates the cellular redox environment and also the activity of certain proteins. In the nucleus, Trx-1 has been shown to interact with many transcription factors such as Ref-1, HIF-1α, NF-κB, p53, AP-1, Nrf-2, glucocorticoid receptor, estrogen receptor, and others [157,167,168,169,170], regulating a large range of gene expression. Intracellular Trx-1 localizes mainly in the cytoplasm. However, several factors induce nuclear translocation of Trx-1, despite the lack of a nuclear localization signal. This nuclear translocation, through karyopherin [168], suggests that Trx-1 may be associated with signaling molecules that bridge the cytoplasmic and nuclear compartments. By contrast, the translocation of Trx-1 to the plasma membrane requires binding of TXNIP. The Trx system is a crucial and essential system for the protection against oxidative stress, for the maintenance of the cellular redox balance, and for the regulation of cell fate [157,171,172].

### 3.2. Truncated Trx, Trx-80

The truncated form of Trx-1, Trx-80, was first termed eosinophil cytotoxicity-enhancing factor (ECEF) due to its eosinophil cytotoxicity, and it was first detected in the plasma of patients suffering from severe schistosomiasis [173,174,175]. Trx-80 (10 kDa) is the product of natural cleavage of Trx-1, sharing the 80 or 84 N-terminal amino acids with Trx-1 [176]. It has been suggested that the enzyme responsible for its cleavage would be an inducible protease [177]. In rheumatoid arthritis (RA), synoviocytes express the truncated form of Trx-80, and treatment with the proinflammatory cytokines IL-1β and/or TNF-*α* increases Trx-80 cell expression, playing an important role in the establishment and/or the development of RA autoimmunity [177].

Gil-Bea et al. have recently shown that the disintegrins and metalloproteinases ADAM-10 and -17, two α-secretases processing the amyloid β precursor protein, are responsible for Trx-80 generation in the brain [178]. Further work is needed to establish other possible candidates in different tissues and under different pathophysiological conditions. Macrophages are capable of cleaving full-length Trx-1 to yield Trx-80, which in contrast to the cytosolic localization of Trx-1, is present mainly at the surface of monocytes [176]. Human brain samples and human primary cultures were shown to produce Trx-80, which polymerizes it into very stable aggregates migrating at approximately 30 kDa in SDS– PAGE [178].

### 3.3. Trx-1 and Trx-80 in Cardiovascular Diseases

Trx-1 system represents an important antioxidant oxidoreductase system involved in a number of clinical conditions [179]. Variable Trx-1 plasma levels have been found in several diseases, such as acute myocardial infarction [180], chronic heart failure [181], carotid atherosclerosis [182], and sepsis [183]. Various studies have reported a protective effect of Trx-1 in CVD context. Trx-1 can improve endothelial function and is able to rescue ECs from age-induced disorders [184]. In addition, vascular Trx-1 prevents eNOS S-glutathionylation, thereby preventing uncoupling of eNOS resulting in improved NO release and decreased oxidative load, consequently maintaining coronary artery perfusion and endothelial function following ischemia-reperfusion injury in mice [185]. Acrolein and HNE are reactive aldehydes generated during active inflammation as a consequence of lipid peroxidation; both react with protein thiols, including Trx-1, which is critical to maintain normal endothelial function and protect against CVDs [186,187]. Acrolein and HNE modify Trx-1 (Cys^72^) in ECs and stimulate inflammatory signaling events, including ROS generation, elevated cell adhesion molecules expression, and increased monocyte adhesion [188]. Chemical modification of Trx-1 by common environmental and endogenously generated reactive aldehydes can contribute to atherosclerosis development by interfering with vasculo-protective functions of Trx1.

In vitro, human recombinant Trx-1 downregulates the expression of a number of inflammatory genes such as IL-1β, TNF-*α*, IL-6, and IL-8 in human macrophages [189]. Moreover, Trx-1 Induces M2 macrophage polarization through downregulation of p16*^INK4a^* and reduces M1 polarization through downregulation of AP-1 and Ref-1. As a consequence, LPS-induced atherosclerotic plaque, in ApoE2-Ki mice, became significantly smaller and more stable [190]. In addition, Trx-1 colocalizes with M2 macrophages in human atherosclerotic lesions [190]. Li W. et al. recently provided evidence that a flavonoid compound, puerarin, activates the Trx-1 redox system, leading to SR-A and Lox-1 reduction and lipid uptake inhibition in macrophages. These results suggest that Trx-1 may serve as target in preventing atherogenesis [191]. However, therapeutic use of Trx-1 is compromised by its in vivo cleavage into Trx-80, according to a not yet described mechanism.

The level of Trx-80 in plasma has been reported to vary from 2 to 175 ng/mL and is markedly increased under inflammatory conditions [192,193]. Trx-80 activates monocytes and induces upregulation of cell surface pathogen recognition receptors, molecules essential for T-cell activation and function [194] and for the release of the proinflammatory cytokines [195]. In contrast to the full-length Trx-1, which downregulates the expression of a number of inflammatory genes [189], Trx-80 promotes mouse peritoneal and human macrophages toward a proinflammatory M1 phenotype, and significantly increases aortic lesion surface area in mice [157]. Trx-80 induced the expression of murine M1 macrophage markers through Akt2/mechanistic target of rapamycin–C1 (mTOR)/70S6K pathway and activated the inflammasome NLRP3, leading to the release of IL-1β and IL-18, potent atherogenic cytokines [196]. Contrarily to Trx-80, Trx-1 activates Akt-1 but not mTOR pathway [196], and inhibits NLRP3 inflammasome [197]. In Apoe^-^−/− mice that overexpress human Trx-80, specifically in macrophages, a significant increase of aortic surface lesion has been described. In addition, in human atherosclerotic plaques, Trx-80 expressed by macrophages colocalized with the M1 macrophage markers [196].

Couchie et al. have shown that the circulating level of the Trx-80 increased in healthy old subjects (>65 years), whereas the level of full-length Trx-1 decreased in the same subjects compared to young individuals (<40 years) [196]. The loss of Trx-1 and the increase of Trx-80 with age appears to be a molecular switch from an anti-inflammatory to a proinflammatory molecule and may contribute, at least in part, to the occurrence of oxidative stress, inflammation, and atherosclerosis in old subjects. The plasma level of Trx-80 could be considered as a new biomarker for the evaluation of inflammaging and the risk of atherosclerotic lesion development in elderly.

## 4. Established and Emerging Therapeutical Strategies for Atherosclerosis

Due to the epidemiological characteristics and importance of atherosclerosis, a huge effort has been made to produce effective therapeutics. Classical therapies such as nutrition, exercise, and statins were first implemented to fight against hypercholesterolemia. The inflammatory nature of atherosclerosis was soon recognized for the development of anti-inflammatory therapeutic strategies targeting its classical risk factors such as dyslipidemia and hypertension. Physical exercise has been demonstrated to be a therapeutic tool for atherosclerosis, however, its beneficial effect is dosage-dependent, and improper over-exercise might also cause damage to the heart [198,199]. Nutraceuticals are natural nutritional compounds (e.g., Omega 3, Vitamin C… etc.) that are beneficial for the prevention or treatment of the disease and, therefore, represent a possible therapeutic avenue for the treatment of atherosclerosis with evidence from in vitro and in vivo studies. However, the main limitation for their use as therapeutics is the challenge of correctly attributing the therapeutic effects to a specific compound, or to a combination of elements [200].

**Figure 3:**
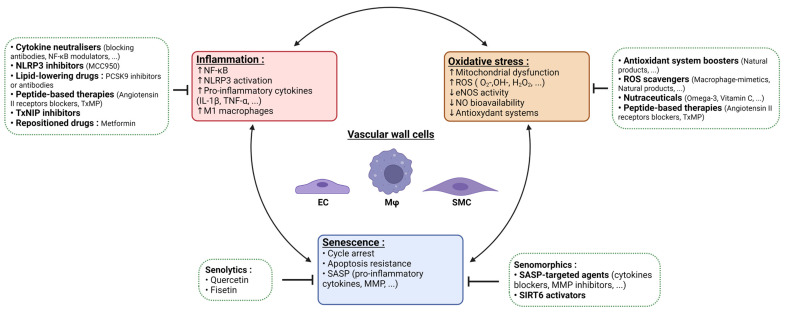
Atherosclerosis triad: targeting inflammatory pathways, oxidative stress, and senescence to derive innovative therapeutic strategies. In atherosclerosis, inflammation, oxidative stress and senescence are interconnected processes (arrows). In this context, main deregulated factors are given for each process together with the principal classes of therapeutic options (green dashed frames), including anti-inflammatory agents (cytokine blockers, inflammasome inhibitors, NF-κB modulators); antioxidants (ROS scavengers, antioxidant system boosters); senolytics; and senormorphics.

Some current and ongoing strategies that focus on the three main targeted hallmarks (inflammation, oxidative stress and senescence) are exemplified below and summarized in Figure 3. 

### 4.1. Anti-Inflammatory and Antioxidant Agents

As presented in preceding sections, inflammation and ROS are central players in the physiopathology of atherosclerosis, particularly modulating macrophage phenotypic plasticity. Drug development aiming to target inflammatory pathways and scavenging ROS or boosting antioxidant systems have been early considered as promising avenues for innovative therapies in atherosclerosis [201,202]. We provide a panoramic survey of established strategies but also give some insights on ongoing options.

The proof of concept that targeting inflammation reduces cardiovascular events in patients with a history of myocardial infarction has highlighted the urgent need to identify new immunotherapies to treat patients with atherosclerotic cardiovascular disease [203]. Immune monitoring in early phases of drug testing was early proposed to advance drug discovery and precision medicine in CVDs to reduce adverse cardiovascular outcomes and death [204]. Among these innovative therapeutics to treat inflammatory components of atherosclerosis, the effects of lipid-lowering drugs on inflammatory biomarkers such as proprotein convertase subtilisin kexin type 9 (PCSK9) inhibitors have been shown to substantially reduce LDL particles and cardiovascular event rates; however, their long-term safety and effects on cardiovascular risk are currently being investigated [205]. However, due to their high price, they remain underutilized and difficult to prescribe in clinical practice [206]. Antibodies that neutralize inflammatory cytokines (TNF-*α*, IL-1β, IL-6, IL-17, and IL-12/23) have shown promising but contradictory results and thus warrant further research [207,208]. Monoclonal antibodies against PCSK9 (alirocumab, evolocumab) were also developed and studied in large clinical programs. These PCSK9 inhibitors lowered plasma LDL levels by approximately 60% [209].

Immune checkpoint proteins have a critical role in facilitating immune cell interactions and play an essential role in the development of atherosclerosis. Immune checkpoints in CVDs have been considered to have the potential to successfully target the residual inflammatory risk that is still present after treatment of classical cardiovascular risk factors [210]. However, several studies report an increased incidence of atherosclerotic CVD after the administration of immune checkpoint inhibitors (ICI), with the occurrence of pathologies such as myocardial infarction, ischemic stroke, and coronary artery disease significantly higher after ICI use [211]. Hence, this is particularly alarming for cancer patients treated with ICI immunotherapies, and not in favor of the development of ICI as potential anti-atherosclerotic drugs.

As mentioned above, NLRP3 inflammasome is a key player in macrophages inflammation and pyroptosis, which is a type of proinflammatory cell-death and takes part in atherosclerotic plaques. The anti-atherosclerotic mechanisms of MCC950, a well-known specific NLRP3 inhibitor, on attenuating macrophages’ inflammation and pyroptosis were reported to be involved in inhibiting the assembly and activation of NLRP3 inflammasome, rather than interrupting its priming [212]. MCC950 was also shown to reduce plaque development, promote plaque stability, and improve vascular function in diabetes-associated vascular disease, thus suggesting that targeting NLRP3-mediated inflammation constitutes an interesting therapeutic strategy [213].

NF-kB is a major signaling pathway in inflammatory processes associated to atherosclerosis. Recently, the pharmacological properties of inosine, an endogenous nucleotide, as NF-kB modulator, administered sub-chronically in a hypercholesterolemic model, have been investigated. It was recently concluded that inosine may be considered as a potential drug for the treatment of cardiovascular disorders such as atherosclerosis [214].

Under oxidative conditions of atherosclerosis, monocytes/macrophages and vascular SMCs highly exposed to oxLDL tend to convert to foam cells due to the intracellular accumulation of lipids [215]. Various options have been proposed to counteract oxidative stress ranging from pharmacological approaches to innovative devices such as macrophage-mimetics [216]. We illustrate below this diversity. ROS from vascular endothelium are strongly related to various enzymes, such as xanthine oxidase, eNOS and Nox. Several pharmaceutical agents, including angiotensin-converting enzyme inhibitors, angiotensin receptors, blockers, and statins, have demonstrated additional antioxidant properties beyond their principal role [217,218].

Thioredoxin mimetic peptides (TxMPs) are considered as powerful bi-functional molecules, harboring both an antioxidant and anti-inflammatory activities [219,220]. We previously demonstrated that one TxMP, CB3, exerts vasculo-protective effects, by reducing inflammation, oxidative stress, NF-κB activation, M1 macrophage orientation, and surfaces of atherosclerotic lesions in ApoE2.Ki mice [221].

Moreover, AVE 0991 is a nonpeptide and orally active angiotensin-(1–7) receptor agonist with an IC_50_ of 21 nm that was also shown to exhibit anti-atherosclerotic and anti-inflammatory actions; affecting monocyte/macrophage differentiation and recruitment to perivascular space during early stages of atherosclerosis in ApoE−/− mice. The study of Skiba et al., also suggested that AVE0991 could serve as valuable alternative to typical immunosuppressants in vascular disease by acting simultaneously on the vasculature and on immune/inflammatory cells [222].

As indicated above, TXNIP is a natural inhibitor of Trx-1 and displays a pivotal for the pathophysiology of various diseases. TXNIP increases ROS production and oxidative stress and thereby contributes to apoptosis. Hence, TXNIP inhibitors also appear as a valuable therapeutic option in atherosclerosis [157,223]. A recent review from Domingues et al. provides a survey of the role of TXNIP in cardiovascular diseases and presents a census of the identified inhibitors of TXNIP [224].

Interestingly, Metformin, a well-known anti- type II diabetes drug, is able to regulate the function of macrophages in atherosclerosis, including reducing the differentiation of monocytes and inhibiting the inflammation, oxidative stress, M1 polarization, foam cell formation, and apoptosis, through for example AMPK, AMPK independent targets, NF-κB, ABCG5/8, Sirt1, FOXO1/FABP4, and HMGB1 [225]. In addition, Metformin was shown to inhibit NLRP3 inflammasome activation, and suppressed atherosclerosis in ApoE−/− mice, at least partially through activation of AMPK and regulation of Trx-1/TxNIP [226].

Many natural products were reported to positively modulate NF-κB pathways via ROS neutralization or by boosting antioxidant systems [202]. Improving antioxidant status through diet was also shown to potentially decrease the progression of atherosclerosis. In particular, anthocyanins, which are flavonoid polyphenols with antioxidant properties, have been associated with reduced risk of cardiovascular disease. Hence, consumption of anthocyanins increases total antioxidant capacity, antioxidant defense enzymes, and HDL antioxidant properties by several measures in preclinical and clinical populations. Anthocyanins were shown to impart antioxidant actions through direct antioxidant properties, as well as indirectly via inducing intracellular Nrf2 activation and antioxidant gene expression. These actions lower oxidative stress and inflammatory signaling in cell populations present in atherosclerotic plaques, including macrophages and ECs [227].

Polydatin, an active ingredient isolated from the natural medicine Polygonum cuspidatum, has been shown to have a prominent role in the treatment of CVDs. Polydatin treats atherosclerosis through three main aspects: anti-inflammatory, regulating lipid metabolism, and anti-oxidative stress [228].

Recently, Kansuinine A (KA), a natural product extracted from *Euphorbia kansui*, was reported as an inhibitor of H_2_O_2_-mediated upregulation of phosphorylated IKKβ, phosphorylated IκBα, and phosphorylated NF-κB. KA also reduced the Bax/Bcl-2 ratio and cleaved caspase-3 expression, preventing H_2_O_2_-induced vascular EC apoptosis, which is of great interest [229]. ROS-sensitive formulations have been widely used in atherosclerosis applications such as ROS scavenging, drug delivery, gene delivery, and imaging [230].

A new biomimetic drug delivery system consisting of nanoparticles that are coated with macrophage membrane and responsive to ROS enables targeted pharmacotherapy for atherosclerosis in mice while also suppressing local inflammation by sequestering inflammatory factors [231]. Hence, macrophage-biomimetic delivery system can achieve inflammation tropism without the need for specific targeting molecules; this strategy might hold promise for other inflammatory diseases [232].

The balance between ROS sensitivity and stability is paramount for enhancing the ultimate efficacy of therapy and imaging and reducing the undesirable side effects of ROS scavenging and antioxidant-enhancing drugs. Further development addressing the key challenges will therefore greatly enhance potential applications of this class of agents in atherosclerosis.

### 4.2. Senomodulating Agents

Targeting inflammation and oxidative stress is a very successful strategy to treat atherosclerosis and related complications. However due to variations in patient responsiveness to treatment owing to the multifactorial nature of atherosclerosis, which results in weak regenerative potential, continuous efforts are needed to provide anti-atherogenic agents with varied mechanisms of action. Targeting SC populations appeared as a valuable strategy since many studies have demonstrated that senescence contributes to the pathophysiology of several age-related including CVDs [233,234]. It was recently reported that senolysis reverses aging phenotype in cardiovascular disorders. Generating therapies targeting elimination of SCs or senoprotective pathways would inhibit the progression of undesirable features of aging, and become promising therapies for CVDs [234].

In line with the multifactorial nature of the disease, senomodulating agents either depleting SC (senolytics) or targeting SASP (senomorphics) appear to be excellent innovative candidates. Quercetin, a senolytic of reference was shown to inhibit the formation of foam cells induced by oxLDL and delay senescence. The mechanism may be related to the regulation of MST1-mediated (mammalian Ste20-like kinase 1) autophagy of RAW264.7 cells [235].

On the other hand, following treatment of ApoE−/− mice with fisetin, another well-known senolytic, and atorvastatin, both the atherosclerotic plaque and the lipid accumulation in the aortic sinus were significantly reduced, and the expressions of PCSK9, LOX-1, and aging markers, including p53, p21, and p16, were downregulated [236]. These treatments, resulting in a multi-pathway effect simultaneously targeting several hallmarks of atherosclerosis seem promising. However, genetic and pharmacological senolysis was shown to have variable effects on atherosclerosis, and may promote inflammation and non-specific effects respectively. In addition, traditional markers of cell senescence such as p16*^INK4A^* have significant limitations in identifying and removing SC in atherosclerosis, suggesting that senescence studies in atherosclerosis and new senolytic drugs require more specific and lineage-restricted markers before ascribing their effects entirely to senolysis [237].

Among the emerging targets of senescence in atherosclerosis, SIRT6 appears as an interesting candidate. Hence, SIRT6 protein expression is reduced in vascular SMC of human and mouse atherosclerotic plaque, and is positively regulated by the ubiquitin ligase CHIP (C terminus of HSC70-interacting protein). SIRT6 regulates telomere maintenance and vascular SMC lifespan, and inhibits atherogenesis, all of which is dependent on its deacetylase activity. Recent data from Bennett and coworkers have shown that endogenous SIRT6 deacetylase is an important and unrecognized inhibitor of SMC senescence and atherosclerosis. It is also worth to note, that this study also supported the possible selective removal of senescent SMCs by novel senolytic drugs, which may slow vascular aging and delay atherosclerosis. Thus, activators of SIRT6 may constitute valuable candidates to prevent pathological VSMC senescence [238].

## 5. Concluding Remarks

During aging, the human mortality rate rises exponentially as a result of a loss of normal organ functions, including tissue maintenance and repair capacity. Moreover, the accumulation of stressors plus the impairment of defense systems efficacy become insurmountable in the elderly, leading to age-related diseases. Age-related cardiovascular disease (CVD), primarily atherosclerosis, is of particular clinical concern as the global population ages, with one billion individuals projected to be over 65 years old by 2030. Aging promotes the development and progression of atherosclerosis through different mechanisms, which are mostly related to age-induced elevations in circulating and intracellular inflammation and oxidative stress as well as to the accumulation of SCs. Given the growing patient population and the inadequacy of current medical management, there is strong incentive to identify new therapeutic targets to treat CVD or, more optimally, prevent it.

Targeting SCs in atherosclerosis as a representative age-related disease is a very encouraging but challenging issue. Heterogeneity within senescent cells is a very critical point to overcome considering that sub-populations of senescent cells namely ECs, vascular SMCs, monocytes, foam cells, and T cells may contribute differentially to the phyisopathological process. Hence, identifying and specifically targeting certain population may represent a unique opportunity to derive new generations of senomodulators; either senolytics or SASP-targeted agents, through the identification of specific markers.

Macrophages are major players of atherosclerosis. Hence, a wide variety of macrophage subtypes with different functions is implicated in the development and progression of atherosclerotic lesions. Their cellular plasticity triggers a variety of phenotypes that play differential roles in the pathology. The best way to counteract the progression of the disease and avoid cardiovascular complications is to intervene at the early stages. Based on huge amounts of data resulting from multiscale and multisystem studies providing new biomarkers and pathways, the emergence of innovative therapeutic strategies is growing. Among them, agents that control cell fate have a high potential to regenerate cardiac tissues from injuries resulting from atherosclerotic plaque ruptures.

Atherosclerosis physiopathogy is based on a triad of processes including inflammation, oxidative stress, and senescence. These processes although considered individually for drug development are intimately linked and open opportunities to derive polypharmacological strategies that simultaneously target overall processes leading to a holistic action. In this context, we propose the thioredoxin system, one of the primordial systems mainly implicated in anti-inflammatory and anti-oxidative defenses. Translation to preclinical and clinical stages is needed to better evaluate their potential as disease modifiers.

**Figure 4:**
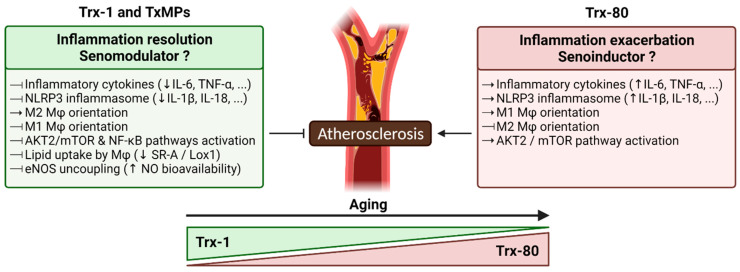
Summary of the principal features of thioredoxin system in atherosclerosis. Truncated thioredoxin (Trx-80) acts as an inflammatory and atherosclerosis inducer while Trx-1 and TxMPs counteract key inflammatory pathways and exert an antiatherogenic effect.

In this review, we place particular emphasis on the thioredoxin system by providing updated information about its implication in atherosclerosis (Figure 4). Indeed, Trx-1 and TxMPs were shown to promote M2 macrophage phenotype and anti-inflammatory pathways by particularly modulating ROS levels, NFκ-B and NLRP3 inflammasome activity, lower lipid uptake by macrophages, and enhancing NO bioavailability. Increased levels of the truncated form of Trx-1, namely Trx-80, were observed in the plasma of aged patients. Moreover, Trx-80 was shown to drive M2 to M1 transition, contributing to an enhanced inflammatory environment particularly by activating NLRP3 inflammasome.

## Data Availability

The data that support the findings of this study are available from the corresponding author upon reasonable request.

## References

[1] Roth G.A., Mensah G.A., Johnson C.O., Addolorato G., Ammirati E., Baddour L.M., Barengo N.C., Beaton A.Z., Benjamin E.J., Benziger C.P. (2020). Global Burden of Cardiovascular Diseases and Risk Factors, 1990–2019. J. Am. Coll. Cardiol..

[2] Madamanchi N.R., Hakim Z.S., Runge M.S. (2005). Oxidative Stress in Atherogenesis and Arterial Thrombosis: The Disconnect between Cellular Studies and Clinical Outcomes. J. Thromb. Haemost..

[3] Libby P., Buring J.E., Badimon L., Hansson G.K., Deanfield J., Bittencourt M.S., Tokgözoğlu L., Lewis E.F. (2019). Atherosclerosis. Nat. Rev. Dis. Primers.

[4] Ji J., Pan E., Li J., Chen J., Cao J., Sun D., Lu X., Chen S., Gu D., Duan X. (2011). Classical Risk Factors of Cardiovascular Disease among Chinese Male Steel Workers: A Prospective Cohort Study for 20 Years. BMC Public Health.

[5] Charles R.L., Burgoyne J.R., Eaton P., Sauer H., Shah A.M., Laurindo F.R.M. (2010). Mechanisms of Redox Signaling in Cardiovascular Disease. Studies on Cardiovascular Disorders.

[6] Hashimoto D., Chow A., Noizat C., Teo P., Beasley M.B., Leboeuf M., Becker C.D., See P., Price J., Lucas D. (2013). Tissue-Resident Macrophages Self-Maintain Locally throughout Adult Life with Minimal Contribution from Circulating Monocytes. Immunity.

[7] Ensan S., Li A., Besla R., Degousee N., Cosme J., Roufaiel M., Shikatani E.A., El-Maklizi M., Williams J.W., Robins L. (2016). Self-Renewing Resident Arterial Macrophages Arise from Embryonic CX3CR1(+) Precursors and Circulating Monocytes Immediately after Birth. Nat. Immunol..

[8] Sena C.M., Pereira A.M., Seiça R. (2013). Endothelial Dysfunction—A Major Mediator of Diabetic Vascular Disease. Biochim. Biophys. Acta.

[9] Bennett M.R., Sinha S., Owens G.K. (2016). Vascular Smooth Muscle Cells in Atherosclerosis. Circ. Res..

[10] Incalza M.A., D’Oria R., Natalicchio A., Perrini S., Laviola L., Giorgino F. (2018). Oxidative Stress and Reactive Oxygen Species in Endothelial Dysfunction Associated with Cardiovascular and Metabolic Diseases. Vascul. Pharmacol..

[11] Liao J.K. (2013). Linking Endothelial Dysfunction with Endothelial Cell Activation. J. Clin. Investig..

[12] Tabas I. (2010). Macrophage Death and Defective Inflammation Resolution in Atherosclerosis. Nat. Rev. Immunol..

[13] Smith E.B., Keen G.A., Grant A. (1990). Factors Influencing the Accumulation in Fibrous Plaques of Lipid Derived from Low Density Lipoprotein. I. Relation between Fibrin and Immobilization of Apo B-Containing Lipoprotein. Atherosclerosis.

[14] Haneklaus M., O’Neill L.A.J. (2015). NLRP3 at the Interface of Metabolism and Inflammation. Immunol. Rev..

[15] Chistiakov D.A., Melnichenko A.A., Myasoedova V.A., Grechko A.V., Orekhov A.N. (2017). Mechanisms of Foam Cell Formation in Atherosclerosis. J. Mol. Med..

[16] Lusis A.J. (2000). Atherosclerosis. Nature.

[17] Glass C.K., Witztum J.L. (2001). Atherosclerosis. The Road Ahead. Cell.

[18] Swirski F.K., Pittet M.J., Kircher M.F., Aikawa E., Jaffer F.A., Libby P., Weissleder R. (2006). Monocyte Accumulation in Mouse Atherogenesis Is Progressive and Proportional to Extent of Disease. Proc. Natl. Acad. Sci. USA.

[19] Williams J.W., Martel C., Potteaux S., Esaulova E., Ingersoll M.A., Elvington A., Saunders B.T., Huang L.-H., Habenicht A.J., Zinselmeyer B.H. (2018). Limited Macrophage Positional Dynamics in Progressing or Regressing Murine Atherosclerotic Plaques-Brief Report. Arterioscler. Thromb. Vasc. Biol..

[20] Nagenborg J., Goossens P., Biessen E.A.L., Donners M.M.P.C. (2017). Heterogeneity of Atherosclerotic Plaque Macrophage Origin, Phenotype and Functions: Implications for Treatment. Eur. J. Pharmacol..

[21] Allahverdian S., Chehroudi A.C., McManus B.M., Abraham T., Francis G.A. (2014). Contribution of Intimal Smooth Muscle Cells to Cholesterol Accumulation and Macrophage-like Cells in Human Atherosclerosis. Circulation.

[22] Libby P., Ridker P.M., Hansson G.K. (2011). Progress and Challenges in Translating the Biology of Atherosclerosis. Nature.

[23] Libby P. (2009). Molecular and Cellular Mechanisms of the Thrombotic Complications of Atherosclerosis. J. Lipid Res..

[24] Libby P. (2002). Atherosclerosis: The New View. Sci. Am..

[25] Virchow R. (1989). As Based upon Physiological and Pathological Histology. Nutr. Rev..

[26] Libby P. (2002). Inflammation in Atherosclerosis. Nature.

[27] Ross R. (1999). Atherosclerosis Is an Inflammatory Disease. Am. Heart J..

[28] Libby P., Okamoto Y., Rocha V.Z., Folco E. (2010). Inflammation in Atherosclerosis: Transition from Theory to Practice. Circ. J..

[29] van Leuven S.I., Franssen R., Kastelein J.J., Levi M., Stroes E.S.G., Tak P.P. (2008). Systemic Inflammation as a Risk Factor for Atherothrombosis. Rheumatology.

[30] Libby P., Loscalzo J., Ridker P.M., Farkouh M.E., Hsue P.Y., Fuster V., Hasan A.A., Amar S. (2018). Inflammation, Immunity, and Infection in Atherothrombosis: JACC Review Topic of the Week. J. Am. Coll. Cardiol..

[31] Libby P. (2021). Inflammation in Atherosclerosis-No Longer a Theory. Clin. Chem..

[32] Ridker P.M. (2016). A Test in Context: High-Sensitivity C-Reactive Protein. J. Am. Coll. Cardiol..

[33] Gimbrone M.A., García-Cardeña G. (2016). Endothelial Cell Dysfunction and the Pathobiology of Atherosclerosis. Circ. Res..

[34] Weber C., Noels H. (2011). Atherosclerosis: Current Pathogenesis and Therapeutic Options. Nat. Med..

[35] Libby P., Ordovas J.M., Birinyi L.K., Auger K.R., Dinarello C.A. (1986). Inducible Interleukin-1 Gene Expression in Human Vascular Smooth Muscle Cells. J. Clin. Investig..

[36] Olivares R., Ducimetière P., Claude J.R. (1993). Monocyte Count: A Risk Factor for Coronary Heart Disease?. Am. J. Epidemiol..

[37] Murphy A.J., Tall A.R. (2016). Disordered Haematopoiesis and Athero-Thrombosis. Eur. Heart J..

[38] Zernecke A., Winkels H., Cochain C., Williams J.W., Wolf D., Soehnlein O., Robbins C.S., Monaco C., Park I., McNamara C.A. (2020). Meta-Analysis of Leukocyte Diversity in Atherosclerotic Mouse Aortas. Circ. Res..

[39] Poznyak A.V., Wu W.-K., Melnichenko A.A., Wetzker R., Sukhorukov V., Markin A.M., Khotina V.A., Orekhov A.N. (2020). Signaling Pathways and Key Genes Involved in Regulation of Foam Cell Formation in Atherosclerosis. Cells.

[40] Abderrazak A., Syrovets T., Couchie D., El Hadri K., Friguet B., Simmet T., Rouis M. (2015). NLRP3 Inflammasome: From a Danger Signal Sensor to a Regulatory Node of Oxidative Stress and Inflammatory Diseases. Redox Biol..

[41] Lehti S., Nguyen S.D., Belevich I., Vihinen H., Heikkilä H.M., Soliymani R., Käkelä R., Saksi J., Jauhiainen M., Grabowski G.A. (2018). Extracellular Lipids Accumulate in Human Carotid Arteries as Distinct Three-Dimensional Structures and Have Proinflammatory Properties. Am. J. Pathol..

[42] Westerterp M., Gautier E.L., Ganda A., Molusky M.M., Wang W., Fotakis P., Wang N., Randolph G.J., D’Agati V.D., Yvan-Charvet L. (2017). Cholesterol Accumulation in Dendritic Cells Links the Inflammasome to Acquired Immunity. Cell Metab..

[43] Rajamäki K., Mäyränpää M.I., Risco A., Tuimala J., Nurmi K., Cuenda A., Eklund K.K., Öörni K., Kovanen P.T. (2016). P38δ MAPK: A Novel Regulator of NLRP3 Inflammasome Activation with Increased Expression in Coronary Atherogenesis. Arterioscler. Thromb. Vasc. Biol..

[44] Van der Heijden T., Kritikou E., Venema W., van Duijn J., van Santbrink P.J., Slütter B., Foks A.C., Bot I., Kuiper J. (2017). NLRP3 Inflammasome Inhibition by MCC950 Reduces Atherosclerotic Lesion Development in Apolipoprotein E-Deficient Mice-Brief Report. Arterioscler. Thromb. Vasc. Biol..

[45] Abderrazak A., Couchie D., Mahmood D.F.D., Elhage R., Vindis C., Laffargue M., Matéo V., Büchele B., Ayala M.R., El Gaafary M. (2015). Anti-Inflammatory and Antiatherogenic Effects of the NLRP3 Inflammasome Inhibitor Arglabin in ApoE2.Ki Mice Fed a High-Fat Diet. Circulation.

[46] Latz E., Xiao T.S., Stutz A. (2013). Activation and Regulation of the Inflammasomes. Nat. Rev. Immunol..

[47] Patel M.N., Carroll R.G., Galván-Peña S., Mills E.L., Olden R., Triantafilou M., Wolf A.I., Bryant C.E., Triantafilou K., Masters S.L. (2017). Inflammasome Priming in Sterile Inflammatory Disease. Trends Mol. Med..

[48] He Y., Hara H., Núñez G. (2016). Mechanism and Regulation of NLRP3 Inflammasome Activation. Trends Biochem. Sci..

[49] Libby P. (2017). Interleukin-1 Beta as a Target for Atherosclerosis Therapy: Biological Basis of CANTOS and Beyond. J. Am. Coll. Cardiol..

[50] Paramel Varghese G., Folkersen L., Strawbridge R.J., Halvorsen B., Yndestad A., Ranheim T., Krohg-Sørensen K., Skjelland M., Espevik T., Aukrust P. (2016). NLRP3 Inflammasome Expression and Activation in Human Atherosclerosis. J. Am. Heart Assoc..

[51] Rajamäki K., Lappalainen J., Oörni K., Välimäki E., Matikainen S., Kovanen P.T., Eklund K.K. (2010). Cholesterol Crystals Activate the NLRP3 Inflammasome in Human Macrophages: A Novel Link between Cholesterol Metabolism and Inflammation. PLoS ONE.

[52] Sheedy F.J., Grebe A., Rayner K.J., Kalantari P., Ramkhelawon B., Carpenter S.B., Becker C.E., Ediriweera H.N., Mullick A.E., Golenbock D.T. (2013). CD36 Coordinates NLRP3 Inflammasome Activation by Facilitating Intracellular Nucleation of Soluble Ligands into Particulate Ligands in Sterile Inflammation. Nat. Immunol..

[53] Duewell P., Latz E. (2013). Assessment and Quantification of Crystal-Induced Lysosomal Damage. Methods Mol. Biol..

[54] Rhoads J.P., Lukens J.R., Wilhelm A.J., Moore J.L., Mendez-Fernandez Y., Kanneganti T.-D., Major A.S. (2017). Oxidized Low-Density Lipoprotein Immune Complex Priming of the Nlrp3 Inflammasome Involves TLR and FcγR Cooperation and Is Dependent on CARD9. J. Immunol..

[55] Grebe A., Hoss F., Latz E. (2018). NLRP3 Inflammasome and the IL-1 Pathway in Atherosclerosis. Circ. Res..

[56] Xiao H., Lu M., Lin T.Y., Chen Z., Chen G., Wang W.-C., Marin T., Shentu T.-P., Wen L., Gongol B. (2013). Sterol Regulatory Element Binding Protein 2 Activation of NLRP3 Inflammasome in Endothelium Mediates Hemodynamic-Induced Atherosclerosis Susceptibility. Circulation.

[57] Abe J., Berk B.C. (2013). Athero-Prone Flow Activation of the SREBP2-NLRP3 Inflammasome Mediates Focal Atherosclerosis. Circulation.

[58] Bevilacqua M.P., Pober J.S., Wheeler M.E., Cotran R.S., Gimbrone M.A. (1985). Interleukin-1 Activation of Vascular Endothelium. Effects on Procoagulant Activity and Leukocyte Adhesion. Am. J. Pathol..

[59] Libby P., Warner S.J., Friedman G.B. (1988). Interleukin 1: A Mitogen for Human Vascular Smooth Muscle Cells That Induces the Release of Growth-Inhibitory Prostanoids. J. Clin. Investig..

[60] Dinarello C.A. (2009). Immunological and Inflammatory Functions of the Interleukin-1 Family. Annu. Rev. Immunol..

[61] Gosselin D., Link V.M., Romanoski C.E., Fonseca G.J., Eichenfield D.Z., Spann N.J., Stender J.D., Chun H.B., Garner H., Geissmann F. (2014). Environment Drives Selection and Function of Enhancers Controlling Tissue-Specific Macrophage Identities. Cell.

[62] Lavin Y., Winter D., Blecher-Gonen R., David E., Keren-Shaul H., Merad M., Jung S., Amit I. (2014). Tissue-Resident Macrophage Enhancer Landscapes Are Shaped by the Local Microenvironment. Cell.

[63] Van den Bossche J., O’Neill L.A., Menon D. (2017). Macrophage Immunometabolism: Where Are We (Going)?. Trends Immunol..

[64] Tabas I., Lichtman A.H. (2017). Monocyte-Macrophages and T Cells in Atherosclerosis. Immunity.

[65] Stöger J.L., Gijbels M.J.J., van der Velden S., Manca M., van der Loos C.M., Biessen E.A.L., Daemen M.J.A.P., Lutgens E., de Winther M.P.J. (2012). Distribution of Macrophage Polarization Markers in Human Atherosclerosis. Atherosclerosis.

[66] Huang W.-C., Sala-Newby G.B., Susana A., Johnson J.L., Newby A.C. (2012). Classical Macrophage Activation Up-Regulates Several Matrix Metalloproteinases through Mitogen Activated Protein Kinases and Nuclear Factor-ΚB. PLoS ONE.

[67] Cochain C., Zernecke A. (2017). Macrophages in Vascular Inflammation and Atherosclerosis. Pflug. Arch..

[68] Bäck M., Yurdagul A., Tabas I., Öörni K., Kovanen P.T. (2019). Inflammation and Its Resolution in Atherosclerosis: Mediators and Therapeutic Opportunities. Nat. Rev. Cardiol..

[69] Shimada K. (2009). Immune System and Atherosclerotic Disease: Heterogeneity of Leukocyte Subsets Participating in the Pathogenesis of Atherosclerosis. Circ. J..

[70] Moore K.J., Tabas I. (2011). Macrophages in the Pathogenesis of Atherosclerosis. Cell.

[71] Miteva K., Madonna R., De Caterina R., Van Linthout S. (2018). Innate and Adaptive Immunity in Atherosclerosis. Vascul. Pharmacol..

[72] Ketelhuth D.F.J., Hansson G.K. (2016). Adaptive Response of T and B Cells in Atherosclerosis. Circ. Res..

[73] Libby P., Hansson G.K. (2015). Inflammation and Immunity in Diseases of the Arterial Tree: Players and Layers. Circ. Res..

[74] Libby P. (2012). Inflammation in Atherosclerosis. Arterioscler. Thromb. Vasc. Biol..

[75] Chinetti-Gbaguidi G., Baron M., Bouhlel M.A., Vanhoutte J., Copin C., Sebti Y., Derudas B., Mayi T., Bories G., Tailleux A. (2011). Human Atherosclerotic Plaque Alternative Macrophages Display Low Cholesterol Handling but High Phagocytosis Because of Distinct Activities of the PPARγ and LXRα Pathways. Circ. Res..

[76] Mosser D.M., Edwards J.P. (2008). Exploring the Full Spectrum of Macrophage Activation. Nat. Rev. Immunol..

[77] Italiani P., Boraschi D. (2014). From Monocytes to M1/M2 Macrophages: Phenotypical vs. Functional Differentiation. Front. Immunol..

[78] Förstermann U., Xia N., Li H. (2017). Roles of Vascular Oxidative Stress and Nitric Oxide in the Pathogenesis of Atherosclerosis. Circ. Res..

[79] Marchio P., Guerra-Ojeda S., Vila J.M., Aldasoro M., Victor V.M., Mauricio M.D. (2019). Targeting Early Atherosclerosis: A Focus on Oxidative Stress and Inflammation. Oxid. Med. Cell. Longev..

[80] Stocker R., Keaney J.F. (2004). Role of Oxidative Modifications in Atherosclerosis. Physiol. Rev..

[81] Niki E. (2018). Oxidant-Specific Biomarkers of Oxidative Stress. Association with Atherosclerosis and Implication for Antioxidant Effects. Free Radic. Biol. Med..

[82] Kattoor A.J., Pothineni N.V.K., Palagiri D., Mehta J.L. (2017). Oxidative Stress in Atherosclerosis. Curr. Atheroscler. Rep..

[83] Landmesser U., Spiekermann S., Preuss C., Sorrentino S., Fischer D., Manes C., Mueller M., Drexler H. (2007). Angiotensin II Induces Endothelial Xanthine Oxidase Activation: Role for Endothelial Dysfunction in Patients with Coronary Disease. Arterioscler. Thromb. Vasc. Biol..

[84] Förstermann U. (2010). Nitric Oxide and Oxidative Stress in Vascular Disease. Pflug. Arch..

[85] Förstermann U. (2008). Oxidative Stress in Vascular Disease: Causes, Defense Mechanisms and Potential Therapies. Nat. Clin. Pract. Cardiovasc. Med..

[86] Mauricio M.D., Guerra-Ojeda S., Marchio P., Valles S.L., Aldasoro M., Escribano-Lopez I., Herance J.R., Rocha M., Vila J.M., Victor V.M. (2018). Nanoparticles in Medicine: A Focus on Vascular Oxidative Stress. Oxid. Med. Cell. Longev..

[87] Poznyak A.V., Grechko A.V., Orekhova V.A., Chegodaev Y.S., Wu W.-K., Orekhov A.N. (2020). Oxidative Stress and Antioxidants in Atherosclerosis Development and Treatment. Biology.

[88] Poljsak B., Šuput D., Milisav I. (2013). Achieving the Balance between ROS and Antioxidants: When to Use the Synthetic Antioxidants. Oxid. Med. Cell. Longev..

[89] Closa D., Folch-Puy E. (2004). Oxygen Free Radicals and the Systemic Inflammatory Response. IUBMB Life.

[90] Chi Z., Melendez A.J. (2007). Role of Cell Adhesion Molecules and Immune-Cell Migration in the Initiation, Onset and Development of Atherosclerosis. Cell Adhes. Migr..

[91] Lassègue B., Griendling K.K. (2010). NADPH Oxidases: Functions and Pathologies in the Vasculature. Arterioscler. Thromb. Vasc. Biol..

[92] Madamanchi N.R., Runge M.S. (2007). Mitochondrial Dysfunction in Atherosclerosis. Circ. Res..

[93] Lee S., Yu S., Park H.J., Jung J., Go G., Kim W. (2019). Rice Bran Oil Ameliorates Inflammatory Responses by Enhancing Mitochondrial Respiration in Murine Macrophages. PLoS ONE.

[94] Salnikova D., Orekhova V., Grechko A., Starodubova A., Bezsonov E., Popkova T., Orekhov A. (2021). Mitochondrial Dysfunction in Vascular Wall Cells and Its Role in Atherosclerosis. Int. J. Mol. Sci..

[95] Corda S., Laplace C., Vicaut E., Duranteau J. (2001). Rapid Reactive Oxygen Species Production by Mitochondria in Endothelial Cells Exposed to Tumor Necrosis Factor-Alpha Is Mediated by Ceramide. Am. J. Respir. Cell Mol. Biol..

[96] Yang D., Elner S.G., Bian Z.-M., Till G.O., Petty H.R., Elner V.M. (2007). Pro-Inflammatory Cytokines Increase Reactive Oxygen Species through Mitochondria and NADPH Oxidase in Cultured RPE Cells. Exp. Eye Res..

[97] Grote K., Flach I., Luchtefeld M., Akin E., Holland S.M., Drexler H., Schieffer B. (2003). Mechanical Stretch Enhances MRNA Expression and Proenzyme Release of Matrix Metalloproteinase-2 (MMP-2) via NAD(P)H Oxidase-Derived Reactive Oxygen Species. Circ. Res..

[98] Sentman M.L., Brännström T., Westerlund S., Laukkanen M.O., Ylä-Herttuala S., Basu S., Marklund S.L. (2001). Extracellular Superoxide Dismutase Deficiency and Atherosclerosis in Mice. Arterioscler. Thromb. Vasc. Biol..

[99] Kirkland J.L. (2016). Translating the Science of Aging into Therapeutic Interventions. Cold Spring Harb. Perspect. Med..

[100] Matthews C., Gorenne I., Scott S., Figg N., Kirkpatrick P., Ritchie A., Goddard M., Bennett M. (2006). Vascular Smooth Muscle Cells Undergo Telomere-Based Senescence in Human Atherosclerosis: Effects of Telomerase and Oxidative Stress. Circ. Res..

[101] Motterle A., Pu X., Wood H., Xiao Q., Gor S., Ng F.L., Chan K., Cross F., Shohreh B., Poston R.N. (2012). Functional Analyses of Coronary Artery Disease Associated Variation on Chromosome 9p21 in Vascular Smooth Muscle Cells. Hum. Mol. Genet..

[102] Holdt L.M., Sass K., Gäbel G., Bergert H., Thiery J., Teupser D. (2011). Expression of Chr9p21 Genes CDKN2B (P15(INK4b)), CDKN2A (P16(INK4a), P14(ARF)) and MTAP in Human Atherosclerotic Plaque. Atherosclerosis.

[103] Erusalimsky J.D., Kurz D.J. (2005). Cellular Senescence in Vivo: Its Relevance in Ageing and Cardiovascular Disease. Exp. Gerontol..

[104] Childs B.G., Baker D.J., Wijshake T., Conover C.A., Campisi J., van Deursen J.M. (2016). Senescent Intimal Foam Cells Are Deleterious at All Stages of Atherosclerosis. Science.

[105] Childs B.G., Li H., van Deursen J.M. (2018). Senescent Cells: A Therapeutic Target for Cardiovascular Disease. J. Clin. Investig..

[106] Stojanović S.D., Fiedler J., Bauersachs J., Thum T., Sedding D.G. (2020). Senescence-Induced Inflammation: An Important Player and Key Therapeutic Target in Atherosclerosis. Eur. Heart J..

[107] Victorelli S., Passos J.F. (2017). Telomeres and Cell Senescence—Size Matters Not. EBioMedicine.

[108] Haycock P.C., Heydon E.E., Kaptoge S., Butterworth A.S., Thompson A., Willeit P. (2014). Leucocyte Telomere Length and Risk of Cardiovascular Disease: Systematic Review and Meta-Analysis. BMJ.

[109] Benetos A., Toupance S., Gautier S., Labat C., Kimura M., Rossi P.M., Settembre N., Hubert J., Frimat L., Bertrand B. (2018). Short Leukocyte Telomere Length Precedes Clinical Expression of Atherosclerosis: The Blood-and-Muscle Model. Circ. Res..

[110] Wang J., Bai Y., Zhao X., Ru J., Kang N., Tian T., Tang L., An Y., Li P. (2018). OxLDL-Mediated Cellular Senescence Is Associated with Increased NADPH Oxidase P47phox Recruitment to Caveolae. Biosci. Rep..

[111] Wang Y.-C., Lee A.-S., Lu L.-S., Ke L.-Y., Chen W.-Y., Dong J.-W., Lu J., Chen Z., Chu C.-S., Chan H.-C. (2018). Human Electronegative LDL Induces Mitochondrial Dysfunction and Premature Senescence of Vascular Cells in Vivo. Aging Cell.

[112] Freund A., Orjalo A.V., Desprez P.-Y., Campisi J. (2010). Inflammatory Networks during Cellular Senescence: Causes and Consequences. Trends Mol. Med..

[113] Westhoff J.H., Hilgers K.F., Steinbach M.P., Hartner A., Klanke B., Amann K., Melk A. (2008). Hypertension Induces Somatic Cellular Senescence in Rats and Humans by Induction of Cell Cycle Inhibitor P16INK4a. Hypertension.

[114] Hernandez-Segura A., Nehme J., Demaria M. (2018). Hallmarks of Cellular Senescence. Trends Cell Biol..

[115] Dimri G.P., Lee X., Basile G., Acosta M., Scott G., Roskelley C., Medrano E.E., Linskens M., Rubelj I., Pereira-Smith O. (1995). A Biomarker That Identifies Senescent Human Cells in Culture and in Aging Skin in Vivo. Proc. Natl. Acad. Sci. USA.

[116] López-Otín C., Blasco M.A., Partridge L., Serrano M., Kroemer G. (2013). The Hallmarks of Aging. Cell.

[117] Muñoz-Espín D., Serrano M. (2014). Cellular Senescence: From Physiology to Pathology. Nat. Rev. Mol. Cell Biol..

[118] Coppé J.-P., Desprez P.-Y., Krtolica A., Campisi J. (2010). The Senescence-Associated Secretory Phenotype: The Dark Side of Tumor Suppression. Annu. Rev. Pathol..

[119] van Deursen J.M. (2014). The Role of Senescent Cells in Ageing. Nature.

[120] Lunyak V.V., Amaro-Ortiz A., Gaur M. (2017). Mesenchymal Stem Cells Secretory Responses: Senescence Messaging Secretome and Immunomodulation Perspective. Front. Genet..

[121] Ohanna M., Giuliano S., Bonet C., Imbert V., Hofman V., Zangari J., Bille K., Robert C., Bressac-de Paillerets B., Hofman P. (2011). Senescent Cells Develop a PARP-1 and Nuclear Factor-{kappa}B-Associated Secretome (PNAS). Genes Dev..

[122] Franceschi C., Bonafè M., Valensin S., Olivieri F., De Luca M., Ottaviani E., De Benedictis G. (2000). Inflamm-Aging. An Evolutionary Perspective on Immunosenescence. Ann. N. Y. Acad. Sci..

[123] Prattichizzo F., De Nigris V., La Sala L., Procopio A.D., Olivieri F., Ceriello A. (2016). “Inflammaging” as a Druggable Target: A Senescence-Associated Secretory Phenotype-Centered View of Type 2 Diabetes. Oxid. Med. Cell. Longev..

[124] Libby P., Ridker P.M., Hansson G.K. (2009). Leducq Transatlantic Network on Atherothrombosis Inflammation in Atherosclerosis: From Pathophysiology to Practice. J. Am. Coll. Cardiol..

[125] Ridker P.M. (2016). From C-Reactive Protein to Interleukin-6 to Interleukin-1: Moving Upstream To Identify Novel Targets for Atheroprotection. Circ. Res..

[126] Tasdemir N., Lowe S.W. (2013). Senescent Cells Spread the Word: Non-Cell Autonomous Propagation of Cellular Senescence. EMBO J..

[127] Libby P. (2016). Assisted Living in the Atheroma: Elderly Macrophages Promote Plaques. Cell Metab..

[128] Baker D.J., Wijshake T., Tchkonia T., LeBrasseur N.K., Childs B.G., van de Sluis B., Kirkland J.L., van Deursen J.M. (2011). Clearance of P16Ink4a-Positive Senescent Cells Delays Ageing-Associated Disorders. Nature.

[129] Baker D.J., Childs B.G., Durik M., Wijers M.E., Sieben C.J., Zhong J., Saltness R., Jeganathan K.B., Versoza G.C., Pezeshki A.-M. (2016). Naturally Occurring P16Ink4a-Positive Cells Shorten Healthy Lifespan. Nature.

[130] Minamino T., Miyauchi H., Yoshida T., Ishida Y., Yoshida H., Komuro I. (2002). Endothelial Cell Senescence in Human Atherosclerosis: Role of Telomere in Endothelial Dysfunction. Circulation.

[131] Minamino T., Komuro I. (2007). Vascular Cell Senescence: Contribution to Atherosclerosis. Circ. Res..

[132] Katsuumi G., Shimizu I., Yoshida Y., Minamino T. (2018). Vascular Senescence in Cardiovascular and Metabolic Diseases. Front. Cardiovasc. Med..

[133] Vasile E., Tomita Y., Brown L.F., Kocher O., Dvorak H.F. (2001). Differential Expression of Thymosin Beta-10 by Early Passage and Senescent Vascular Endothelium Is Modulated by VPF/VEGF: Evidence for Senescent Endothelial Cells in Vivo at Sites of Atherosclerosis. FASEB J..

[134] Wang J., Uryga A.K., Reinhold J., Figg N., Baker L., Finigan A., Gray K., Kumar S., Clarke M., Bennett M. (2015). Vascular Smooth Muscle Cell Senescence Promotes Atherosclerosis and Features of Plaque Vulnerability. Circulation.

[135] Alloza I., Goikuria H., Idro J.L., Triviño J.C., Fernández Velasco J.M., Elizagaray E., García-Barcina M., Montoya-Murillo G., Sarasola E., Vega Manrique R. (2017). RNAseq Based Transcriptomics Study of SMCs from Carotid Atherosclerotic Plaque: BMP2 and IDs Proteins Are Crucial Regulators of Plaque Stability. Sci. Rep..

[136] Sanada F., Muratsu J., Otsu R., Shimizu H., Koibuchi N., Uchida K., Taniyama Y., Yoshimura S., Rakugi H., Morishita R. (2017). Local Production of Activated Factor X in Atherosclerotic Plaque Induced Vascular Smooth Muscle Cell Senescence. Sci. Rep..

[137] Lähteenvuo J., Rosenzweig A. (2012). Effects of Aging on Angiogenesis. Circ. Res..

[138] Sedding D.G., Boyle E.C., Demandt J.A.F., Sluimer J.C., Dutzmann J., Haverich A., Bauersachs J. (2018). Vasa Vasorum Angiogenesis: Key Player in the Initiation and Progression of Atherosclerosis and Potential Target for the Treatment of Cardiovascular Disease. Front. Immunol..

[139] Boyle E.C., Sedding D.G., Haverich A. (2017). Targeting Vasa Vasorum Dysfunction to Prevent Atherosclerosis. Vascul. Pharmacol..

[140] Sato K., Park N.G., Kohno T., Maeda T., Kim J.I., Kato R., Takahashi M. (1993). Role of Basic Residues for the Binding of Omega-Conotoxin GVIA to N-Type Calcium Channels. Biochem. Biophys. Res. Commun..

[141] Hayashi T., Yano K., Matsui-Hirai H., Yokoo H., Hattori Y., Iguchi A. (2008). Nitric Oxide and Endothelial Cellular Senescence. Pharmacol. Ther..

[142] Uryga A.K., Bennett M.R. (2016). Ageing Induced Vascular Smooth Muscle Cell Senescence in Atherosclerosis. J. Physiol..

[143] Bennett M.R., Evan G.I., Schwartz S.M. (1995). Apoptosis of Human Vascular Smooth Muscle Cells Derived from Normal Vessels and Coronary Atherosclerotic Plaques. J. Clin. Investig..

[144] Song Y., Shen H., Schenten D., Shan P., Lee P.J., Goldstein D.R. (2012). Aging Enhances the Basal Production of IL-6 and CCL2 in Vascular Smooth Muscle Cells. Arterioscler. Thromb. Vasc. Biol..

[145] Gardner S.E., Humphry M., Bennett M.R., Clarke M.C.H. (2015). Senescent Vascular Smooth Muscle Cells Drive Inflammation Through an Interleukin-1α-Dependent Senescence-Associated Secretory Phenotype. Arterioscler. Thromb. Vasc. Biol..

[146] Burton D.G.A., Matsubara H., Ikeda K. (2010). Pathophysiology of Vascular Calcification: Pivotal Role of Cellular Senescence in Vascular Smooth Muscle Cells. Exp. Gerontol..

[147] Wang M., Kim S.H., Monticone R.E., Lakatta E.G. (2015). Matrix Metalloproteinases Promote Arterial Remodeling in Aging, Hypertension, and Atherosclerosis. Hypertension.

[148] Hudgins A.D., Tazearslan C., Tare A., Zhu Y., Huffman D., Suh Y. (2018). Age- and Tissue-Specific Expression of Senescence Biomarkers in Mice. Front. Genet..

[149] Chi C., Li D.-J., Jiang Y.-J., Tong J., Fu H., Wu Y.-H., Shen F.-M. (2019). Vascular Smooth Muscle Cell Senescence and Age-Related Diseases: State of the Art. Biochim. Biophys. Acta (BBA)—Mol. Basis Dis..

[150] Solana R., Pawelec G., Tarazona R. (2006). Aging and Innate Immunity. Immunity.

[151] Weng N.-P. (2006). Aging of the Immune System: How Much Can the Adaptive Immune System Adapt?. Immunity.

[152] Gomez C.R., Boehmer E.D., Kovacs E.J. (2005). The Aging Innate Immune System. Curr. Opin. Immunol..

[153] Ginaldi L., Loreto M.F., Corsi M.P., Modesti M., De Martinis M. (2001). Immunosenescence and Infectious Diseases. Microbes Infect..

[154] Hall B.M., Balan V., Gleiberman A.S., Strom E., Krasnov P., Virtuoso L.P., Rydkina E., Vujcic S., Balan K., Gitlin I.I. (2017). P16(Ink4a) and Senescence-Associated β-Galactosidase Can Be Induced in Macrophages as Part of a Reversible Response to Physiological Stimuli. Aging.

[155] Hall B.M., Balan V., Gleiberman A.S., Strom E., Krasnov P., Virtuoso L.P., Rydkina E., Vujcic S., Balan K., Gitlin I. (2016). Aging of Mice Is Associated with P16(Ink4a)- and β-Galactosidase-Positive Macrophage Accumulation That Can Be Induced in Young Mice by Senescent Cells. Aging.

[156] Laurent T.C., Moore E.C., Reichard P. (1964). Enzymatic synthesis of deoxyribonucleotides: IV. Isolation and characterization of thioredoxin, the hydrogen donor from *Escherichia coli* B. J. Biol. Chem..

[157] Mahmood D.F.D., Abderrazak A., El Hadri K., Simmet T., Rouis M. (2013). The Thioredoxin System as a Therapeutic Target in Human Health and Disease. Antioxid. Redox Signal..

[158] Nordberg J., Arnér E.S. (2001). Reactive Oxygen Species, Antioxidants, and the Mammalian Thioredoxin System. Free Radic. Biol. Med..

[159] Gromer S., Urig S., Becker K. (2004). The Thioredoxin System—From Science to Clinic. Med. Res. Rev..

[160] Lu J., Holmgren A. (2014). The Thioredoxin Antioxidant System. Free Radic. Biol. Med..

[161] Nishiyama A., Matsui M., Iwata S., Hirota K., Masutani H., Nakamura H., Takagi Y., Sono H., Gon Y., Yodoi J. (1999). Identification of Thioredoxin-Binding Protein-2/Vitamin D(3) up-Regulated Protein 1 as a Negative Regulator of Thioredoxin Function and Expression. J. Biol. Chem..

[162] Arnér E.S., Holmgren A. (2000). Physiological Functions of Thioredoxin and Thioredoxin Reductase. Eur. J. Biochem..

[163] Fu C., Wu C., Liu T., Ago T., Zhai P., Sadoshima J., Li H. (2009). Elucidation of Thioredoxin Target Protein Networks in Mouse. Mol. Cell Proteom..

[164] Kim Y.C., Masutani H., Yamaguchi Y., Itoh K., Yamamoto M., Yodoi J. (2001). Hemin-Induced Activation of the Thioredoxin Gene by Nrf2. A Differential Regulation of the Antioxidant Responsive Element by a Switch of Its Binding Factors. J. Biol. Chem..

[165] Chen Z.-H., Saito Y., Yoshida Y., Sekine A., Noguchi N., Niki E. (2005). 4-Hydroxynonenal Induces Adaptive Response and Enhances PC12 Cell Tolerance Primarily through Induction of Thioredoxin Reductase 1 via Activation of Nrf2. J. Biol. Chem..

[166] Harding H.P., Zhang Y., Ron D. (1999). Protein Translation and Folding Are Coupled by an Endoplasmic-Reticulum-Resident Kinase. Nature.

[167] Schenk H., Klein M., Erdbrügger W., Dröge W., Schulze-Osthoff K. (1994). Distinct Effects of Thioredoxin and Antioxidants on the Activation of Transcription Factors NF-Kappa B and AP-1. Proc. Natl. Acad. Sci. USA.

[168] Schroeder P., Popp R., Wiegand B., Altschmied J., Haendeler J. (2007). Nuclear Redox-Signaling Is Essential for Apoptosis Inhibition in Endothelial Cells--Important Role for Nuclear Thioredoxin-1. Arterioscler. Thromb. Vasc. Biol..

[169] Hirota K., Matsui M., Iwata S., Nishiyama A., Mori K., Yodoi J. (1997). AP-1 Transcriptional Activity Is Regulated by a Direct Association between Thioredoxin and Ref-1. Proc. Natl. Acad. Sci. USA.

[170] Hirota K., Murata M., Sachi Y., Nakamura H., Takeuchi J., Mori K., Yodoi J. (1999). Distinct Roles of Thioredoxin in the Cytoplasm and in the Nucleus. A Two-Step Mechanism of Redox Regulation of Transcription Factor NF-KappaB. J. Biol. Chem..

[171] Lu J., Holmgren A. (2012). Thioredoxin System in Cell Death Progression. Antioxid. Redox Signal..

[172] Nishiyama A., Masutani H., Nakamura H., Nishinaka Y., Yodoi J. (2001). Redox Regulation by Thioredoxin and Thioredoxin-Binding Proteins. IUBMB Life.

[173] Dessein A.J., Lenzi H.L., Bina J.C., Carvalho E.M., Weiser W.Y., Andrade Z.A., David J.R. (1984). Modulation of Eosinophil Cytotoxicity by Blood Mononuclear Cells from Healthy Subjects and Patients with Chronic Schistosomiasis Mansoni. Cell. Immunol..

[174] Lenzi H.L., Mednis A.D., Dessein A.J. (1985). Activation of Human Eosinophils by Monokines and Lymphokines: Source and Biochemical Characteristics of the Eosinophil Cytotoxicity-Enhancing Activity Produced by Blood Mononuclear Cells. Cell. Immunol..

[175] Silberstein D.S., Ali M.H., Baker S.L., David J.R. (1989). Human Eosinophil Cytotoxicity-Enhancing Factor. Purification, Physical Characteristics, and Partial Amino Acid Sequence of an Active Polypeptide. J. Immunol..

[176] Pekkari K., Holmgren A. (2004). Truncated Thioredoxin: Physiological Functions and Mechanism. Antioxid. Redox Signal..

[177] Lemarechal H., Anract P., Beaudeux J.-L., Bonnefont-Rousselot D., Ekindjian O.G., Borderie D. (2007). Expression and Extracellular Release of Trx80, the Truncated Form of Thioredoxin, by TNF-Alpha- and IL-1beta-Stimulated Human Synoviocytes from Patients with Rheumatoid Arthritis. Clin. Sci..

[178] Gil-Bea F., Akterin S., Persson T., Mateos L., Sandebring A., Avila-Cariño J., Gutierrez-Rodriguez A., Sundström E., Holmgren A., Winblad B. (2012). Thioredoxin-80 Is a Product of Alpha-Secretase Cleavage That Inhibits Amyloid-Beta Aggregation and Is Decreased in Alzheimer’s Disease Brain. EMBO Mol. Med..

[179] Becker K., Gromer S., Schirmer R.H., Müller S. (2000). Thioredoxin Reductase as a Pathophysiological Factor and Drug Target. Eur. J. Biochem..

[180] Miyamoto S., Sakamoto T., Soejima H., Shimomura H., Kajiwara I., Kojima S., Hokamaki J., Sugiyama S., Yoshimura M., Ozaki Y. (2003). Plasma Thioredoxin Levels and Platelet Aggregability in Patients with Acute Myocardial Infarction. Am. Heart J..

[181] Jekell A., Hossain A., Alehagen U., Dahlström U., Rosén A. (2004). Elevated Circulating Levels of Thioredoxin and Stress in Chronic Heart Failure. Eur. J. Heart Fail..

[182] Madrigal-Matute J., Fernandez-Garcia C.-E., Blanco-Colio L.M., Burillo E., Fortuño A., Martinez-Pinna R., Llamas-Granda P., Beloqui O., Egido J., Zalba G. (2015). Thioredoxin-1/Peroxiredoxin-1 as Sensors of Oxidative Stress Mediated by NADPH Oxidase Activity in Atherosclerosis. Free Radic. Biol. Med..

[183] Leaver S.K., MacCallum N.S., Pingle V., Hacking M.B., Quinlan G.J., Evans T.W., Burke-Gaffney A. (2010). Increased Plasma Thioredoxin Levels in Patients with Sepsis: Positive Association with Macrophage Migration Inhibitory Factor. Intensive Care Med..

[184] Altschmied J., Haendeler J. (2009). Thioredoxin-1 and Endothelial Cell Aging: Role in Cardiovascular Diseases. Antioxid. Redox Signal..

[185] Subramani J., Kundumani-Sridharan V., Hilgers R.H.P., Owens C., Das K.C. (2016). Thioredoxin Uses a GSH-Independent Route to Deglutathionylate Endothelial Nitric-Oxide Synthase and Protect against Myocardial Infarction. J. Biol. Chem..

[186] Yamawaki H., Haendeler J., Berk B.C. (2003). Thioredoxin: A Key Regulator of Cardiovascular Homeostasis. Circ. Res..

[187] Haendeler J., Hoffmann J., Zeiher A.M., Dimmeler S. (2004). Antioxidant Effects of Statins via S-Nitrosylation and Activation of Thioredoxin in Endothelial Cells: A Novel Vasculoprotective Function of Statins. Circulation.

[188] Go Y.-M., Halvey P.J., Hansen J.M., Reed M., Pohl J., Jones D.P. (2007). Reactive Aldehyde Modification of Thioredoxin-1 Activates Early Steps of Inflammation and Cell Adhesion. Am. J. Pathol..

[189] Billiet L., Furman C., Larigauderie G., Copin C., Brand K., Fruchart J.-C., Rouis M. (2005). Extracellular Human Thioredoxin-1 Inhibits Lipopolysaccharide-Induced Interleukin-1beta Expression in Human Monocyte-Derived Macrophages. J. Biol. Chem..

[190] El Hadri K., Mahmood D.F.D., Couchie D., Jguirim-Souissi I., Genze F., Diderot V., Syrovets T., Lunov O., Simmet T., Rouis M. (2012). Thioredoxin-1 Promotes Anti-Inflammatory Macrophages of the M2 Phenotype and Antagonizes Atherosclerosis. Arterioscler. Thromb. Vasc. Biol..

[191] Li W., Xu X., Dong D., Lei T., Ou H. (2021). Up-Regulation of Thioredoxin System by Puerarin Inhibits Lipid Uptake in Macrophages. Free Radic. Biol. Med..

[192] Cortes-Bratti X., Bassères E., Herrera-Rodriguez F., Botero-Kleiven S., Coppotelli G., Andersen J.B., Masucci M.G., Holmgren A., Chaves-Olarte E., Frisan T. (2011). Thioredoxin 80-Activated-Monocytes (TAMs) Inhibit the Replication of Intracellular Pathogens. PLoS ONE.

[193] Pekkari K., Gurunath R., Arner E.S., Holmgren A. (2000). Truncated Thioredoxin Is a Mitogenic Cytokine for Resting Human Peripheral Blood Mononuclear Cells and Is Present in Human Plasma. J. Biol. Chem..

[194] Lemarechal H., Anract P., Beaudeux J.-L., Bonnefont-Rousselot D., Ekindjian O.G., Borderie D. (2007). Impairment of Thioredoxin Reductase Activity by Oxidative Stress in Human Rheumatoid Synoviocytes. Free Radic. Res..

[195] Bertini R., Howard O.M., Dong H.F., Oppenheim J.J., Bizzarri C., Sergi R., Caselli G., Pagliei S., Romines B., Wilshire J.A. (1999). Thioredoxin, a Redox Enzyme Released in Infection and Inflammation, Is a Unique Chemoattractant for Neutrophils, Monocytes, and T Cells. J. Exp. Med..

[196] Couchie D., Vaisman B., Abderrazak A., Mahmood D.F.D., Hamza M.M., Canesi F., Diderot V., El Hadri K., Nègre-Salvayre A., Le Page A. (2017). Human Plasma Thioredoxin-80 Increases With Age and in ApoE−/− Mice Induces Inflammation, Angiogenesis, and Atherosclerosis. Circulation.

[197] Wang Y., Ji N., Gong X., Ni S., Xu L., Zhang H. (2020). Thioredoxin-1 Attenuates Atherosclerosis Development through Inhibiting NLRP3 Inflammasome. Endocrine.

[198] Yang J., Cao R.Y., Gao R., Mi Q., Dai Q., Zhu F. (2017). Physical Exercise Is a Potential “Medicine” for Atherosclerosis. Adv. Exp. Med. Biol..

[199] Lim R.M.H., Koh A.S. (2021). Cardiovascular Aging and Physical Activity: Insights From Metabolomics. Front. Cardiovasc. Med..

[200] Moss J.W.E., Ramji D.P. (2016). Nutraceutical Therapies for Atherosclerosis. Nat. Rev. Cardiol..

[201] Bertrand M.-J., Tardif J.-C. (2017). Inflammation and beyond: New Directions and Emerging Drugs for Treating Atherosclerosis. Expert Opin. Emerg. Drugs.

[202] Malekmohammad K., Sewell R.D.E., Rafieian-Kopaei M. (2019). Antioxidants and Atherosclerosis: Mechanistic Aspects. Biomolecules.

[203] Fernandez D.M., Giannarelli C. (2021). Immune Cell Profiling in Atherosclerosis: Role in Research and Precision Medicine. Nat. Rev. Cardiol..

[204] Charo I.F., Taub R. (2011). Anti-Inflammatory Therapeutics for the Treatment of Atherosclerosis. Nat. Rev. Drug Discov..

[205] Pradhan A.D., Aday A.W., Rose L.M., Ridker P.M. (2018). Residual Inflammatory Risk on Treatment With PCSK9 Inhibition and Statin Therapy. Circulation.

[206] Smith A., Johnson D., Banks J., Keith S.W., Karalis D.G. (2021). Trends in PCSK9 Inhibitor Prescriptions before and after the Price Reduction in Patients with Atherosclerotic Cardiovascular Disease. JCM.

[207] Ji E., Lee S. (2021). Antibody-Based Therapeutics for Atherosclerosis and Cardiovascular Diseases. IJMS.

[208] Chen B., Shi X., Cui Y., Hou A., Zhao P. (2019). A Review of PCSK9 Inhibitors and Their Effects on Cardiovascular Diseases. Curr. Top. Med. Chem..

[209] Sabatine M.S. (2019). PCSK9 Inhibitors: Clinical Evidence and Implementation. Nat. Rev. Cardiol..

[210] Kusters P.J.H., Lutgens E., Seijkens T.T.P. (2018). Exploring Immune Checkpoints as Potential Therapeutic Targets in Atherosclerosis. Cardiovasc. Res..

[211] Poels K., Neppelenbroek S.I.M., Kersten M.J., Antoni M.L., Lutgens E., Seijkens T.T.P. (2021). Immune Checkpoint Inhibitor Treatment and Atherosclerotic Cardiovascular Disease: An Emerging Clinical Problem. J. Immunother. Cancer.

[212] Zeng W., Wu D., Sun Y., Suo Y., Yu Q., Zeng M., Gao Q., Yu B., Jiang X., Wang Y. (2021). The Selective NLRP3 Inhibitor MCC950 Hinders Atherosclerosis Development by Attenuating Inflammation and Pyroptosis in Macrophages. Sci. Rep..

[213] Sharma A., Choi J.S.Y., Stefanovic N., Al-Sharea A., Simpson D.S., Mukhamedova N., Jandeleit-Dahm K., Murphy A.J., Sviridov D., Vince J.E. (2021). Specific NLRP3 Inhibition Protects Against Diabetes-Associated Atherosclerosis. Diabetes.

[214] Lima G.F., de Oliveira Lopes R., Mendes A.B.A., Brazão S.C., Autran L.J., Motta N.A.V., Brito F.C.F. (2020). Inosine, an Endogenous Purine Nucleoside, Avoids Early Stages of Atherosclerosis Development Associated to ENOS Activation and P38 MAPK/NF-KB Inhibition in Rats. Eur. J. Pharmacol..

[215] Zingg J.-M., Vlad A., Ricciarelli R. (2021). Oxidized LDLs as Signaling Molecules. Antioxidants.

[216] Forman H.J., Zhang H. (2021). Targeting Oxidative Stress in Disease: Promise and Limitations of Antioxidant Therapy. Nat. Rev. Drug Discov..

[217] Tousoulis D., Psaltopoulou T., Androulakis E., Papageorgiou N., Papaioannou S., Oikonomou E., Synetos A., Stefanadis C. (2015). Oxidative Stress and Early Atherosclerosis: Novel Antioxidant Treatment. Cardiovasc. Drugs Ther..

[218] Kontoghiorghes G.J., Kontoghiorghe C.N. (2019). Prospects for the Introduction of Targeted Antioxidant Drugs for the Prevention and Treatment of Diseases Related to Free Radical Pathology. Expert Opin. Investig. Drugs.

[219] Atlas D. (2021). Emerging Therapeutic Opportunities of Novel Thiol-Amides, NAC-Amide (AD4/NACA) and Thioredoxin Mimetics (TXM-Peptides) for Neurodegenerative-Related Disorders. Free Radic. Biol. Med..

[220] Lejnev K., Khomsky L., Bokvist K., Mistriel-Zerbib S., Naveh T., Farb T.B., Alsina-Fernandez J., Atlas D. (2016). Thioredoxin-Mimetic Peptides (TXM) Inhibit Inflammatory Pathways Associated with High-Glucose and Oxidative Stress. Free Radic. Biol. Med..

[221] Canesi F., Mateo V., Couchie D., Karabina S., Nègre-Salvayre A., Rouis M., El Hadri K. (2019). A Thioredoxin-Mimetic Peptide Exerts Potent Anti-Inflammatory, Antioxidant, and Atheroprotective Effects in ApoE2.Ki Mice Fed High Fat Diet. Cardiovasc. Res..

[222] Skiba D.S., Nosalski R., Mikolajczyk T.P., Siedlinski M., Rios F.J., Montezano A.C., Jawien J., Olszanecki R., Korbut R., Czesnikiewicz-Guzik M. (2017). Anti-Atherosclerotic Effect of the Angiotensin 1-7 Mimetic AVE0991 Is Mediated by Inhibition of Perivascular and Plaque Inflammation in Early Atherosclerosis. Br. J. Pharmacol..

[223] Cao X., He W., Pang Y., Cao Y., Qin A. (2020). Redox-Dependent and Independent Effects of Thioredoxin Interacting Protein. Biol. Chem..

[224] Domingues A., Jolibois J., Marquet de Rougé P., Nivet-Antoine V. (2021). The Emerging Role of TXNIP in Ischemic and Cardiovascular Diseases; A Novel Marker and Therapeutic Target. Int. J. Mol. Sci..

[225] Feng X., Chen W., Ni X., Little P.J., Xu S., Tang L., Weng J. (2021). Metformin, Macrophage Dysfunction and Atherosclerosis. Front. Immunol..

[226] Tang G., Duan F., Li W., Wang Y., Zeng C., Hu J., Li H., Zhang X., Chen Y., Tan H. (2019). Metformin Inhibited Nod-like Receptor Protein 3 Inflammasomes Activation and Suppressed Diabetes-Accelerated Atherosclerosis in ApoE−/− Mice. Biomed. Pharmacother..

[227] Garcia C., Blesso C.N. (2021). Antioxidant Properties of Anthocyanins and Their Mechanism of Action in Atherosclerosis. Free Radic. Biol. Med..

[228] Wu M., Li X., Wang S., Yang S., Zhao R., Xing Y., Liu L. (2020). Polydatin for Treating Atherosclerotic Diseases: A Functional and Mechanistic Overview. Biomed. Pharmacother..

[229] Chen C.-S., Pan B.-Y., Tsai P.-H., Chen F.-Y., Yang W.-C., Shen M.-Y. (2021). Kansuinine A Ameliorates Atherosclerosis and Human Aortic Endothelial Cell Apoptosis by Inhibiting Reactive Oxygen Species Production and Suppressing IKKβ/IκBα/NF-ΚB Signaling. Int. J. Mol. Sci..

[230] Ji H., Peng R., Jin L., Ma J., Yang Q., Sun D., Wu W. (2021). Recent Advances in ROS-Sensitive Nano-Formulations for Atherosclerosis Applications. Pharmaceutics.

[231] Gao C., Huang Q., Liu C., Kwong C.H.T., Yue L., Wan J.-B., Lee S.M.Y., Wang R. (2020). Treatment of Atherosclerosis by Macrophage-Biomimetic Nanoparticles via Targeted Pharmacotherapy and Sequestration of Proinflammatory Cytokines. Nat. Commun..

[232] Tang D., Wang Y., Wijaya A., Liu B., Maruf A., Wang J., Xu J., Liao X., Wu W., Wang G. (2021). ROS-Responsive Biomimetic Nanoparticles for Potential Application in Targeted Anti-Atherosclerosis. Regen. Biomater..

[233] Lagoumtzi S.M., Chondrogianni N. (2021). Senolytics and Senomorphics: Natural and Synthetic Therapeutics in the Treatment of Aging and Chronic Diseases. Free Radic. Biol. Med..

[234] Owens W.A., Walaszczyk A., Spyridopoulos I., Dookun E., Richardson G.D. (2021). Senescence and Senolytics in Cardiovascular Disease: Promise and Potential Pitfalls. Mech. Ageing Dev..

[235] Cao H., Jia Q., Yan L., Chen C., Xing S., Shen D. (2019). Quercetin Suppresses the Progression of Atherosclerosis by Regulating MST1-Mediated Autophagy in Ox-LDL-Induced RAW264.7 Macrophage Foam Cells. Int. J. Mol. Sci..

[236] Yan L., Jia Q., Cao H., Chen C., Xing S., Huang Y., Shen D. (2021). Fisetin Ameliorates Atherosclerosis by Regulating PCSK9 and LOX-1 in ApoE−/− Mice. Exp. Ther. Med..

[237] Ma S., Fan L., Cao F. (2019). Combating Cellular Senescence by Sirtuins: Implications for Atherosclerosis. Biochim. Biophys. Acta Mol. Basis Dis..

[238] Grootaert M.O.J., Finigan A., Figg N.L., Uryga A.K., Bennett M.R. (2021). SIRT6 Protects Smooth Muscle Cells From Senescence and Reduces Atherosclerosis. Circ. Res..

